# Followers do not dictate the virality of news outlets on social media

**DOI:** 10.1093/pnasnexus/pgae257

**Published:** 2024-06-28

**Authors:** Emanuele Sangiorgio, Matteo Cinelli, Roy Cerqueti, Walter Quattrociocchi

**Affiliations:** Department of Social Sciences and Economics, Sapienza University of Rome, Rome 00185, Italy; Department of Computer Science, Sapienza University of Rome, Rome 00161, Italy; Department of Social Sciences and Economics, Sapienza University of Rome, Rome 00185, Italy; GRANEM, Université d’Angers, SFR Confluences, Angers F-49000, France; Department of Computer Science, Sapienza University of Rome, Rome 00161, Italy

**Keywords:** social media, growth dynamics, attention economy

## Abstract

Initially conceived for entertainment, social media platforms have profoundly transformed the dissemination of information and consequently reshaped the dynamics of agenda-setting. In this scenario, understanding the factors that capture audience attention and drive viral content is crucial. Employing Gibrat’s Law, which posits that an entity’s growth rate is unrelated to its size, we examine the engagement growth dynamics of news outlets on social media. Our analysis includes the Facebook historical data of over a thousand news outlets, encompassing approximately 57 million posts in four European languages from 2008 to the end of 2022. We discover universal growth dynamics according to which news virality is independent of the traditional size of the outlet. Moreover, our analysis reveals a significant long-term impact of news source reliability on engagement growth, with engagement induced by unreliable sources decreasing over time. We conclude the article by presenting a statistical model replicating the observed growth dynamics.

**Significance Statement**

Originally intended for entertainment, social media platforms have profoundly transformed agenda-setting dynamics. Our massive 15-year Facebook analysis of 1,082 news outlets and 57 million posts challenges the assumption that larger news outlets naturally have a greater virality potential. We introduce a model mirroring these dynamics, indicating that virality is influenced more by consistent random processes than by outlet prominence. Notably, we identify a pivotal influence of news source reliability on long-term engagement growth, revealing that trustworthiness affects audience interactions, especially as misinformation challenges platforms. Together, these insights call for a nuanced recognition of information sources, emphasizing that content holds considerable power in shaping public discourse regardless of its origin.

## Introduction

Originally designed for entertainment, social media platforms have evolved into significant channels for information dissemination (1–6), altering traditional agenda-setting dynamics (7–10). In this competitive landscape marked by many information sources, we aim to uncover the determinants of audience attention and the factors contributing to content virality (11), that is the propensity of content to achieve rapid diffusion and disproportionate engagement levels on social media platforms (12–14). Indeed, social media often dictate which topics become prominent while others are overlooked (10, 15). As online users tend to favor information aligning with their existing beliefs, commonly ignoring opposing perspectives (16–19), pieces of content and sources act as a way to justify and offer rationales for their viewpoints. This behavior can create and reinforce online “echo chambers” (20)—digital clusters of homogeneous thought where narratives are collectively shaped and solidified (21–23). The magnitude of the echo chamber phenomenon and its consequent effects on polarization may vary among social media platforms (24). Furthermore, many platforms implement algorithms designed to prioritize user engagement that might alter information spreading (25–27), thereby exacerbating ideological divisions (28–30). The rise of the attention economy is at the heart of digital discourse transformation (31–34). In this economy, a broad spectrum of content creators, ranging from news outlets to individual influencers, vie for limited users’ attention (35–38). Like traditional market evolution, digital stakeholders chase user engagement, converting this captured attention into tangible revenues through advertising, service offerings, and subscription models (39, 40). With revenues closely linked to audience reach and engagement (41–44), understanding the growth mechanisms of digital content creators is crucial.

Our research aims to unravel the dynamics of the digital ecosystem, focusing on the evolution of content consumption and audience reach. We anchor our analysis in Gibrat’s Law (45), originally formulated to explain traditional business growth, extending its application to the digital domain. The foundational premise of this law, positing that a firm’s growth rate is independent of its initial size, has found evidence across decades (46–49) and induced various insights, from explaining the formation of skewed distributions of firms’ sizes (50–52) to the stochastic modeling of companies growth (53–55). In the business field, Gibrat’s Law has been broadly tested with different samples and methodologies, yielding mixed results, from rejection (56–58) to confirmation (59–61). One significant finding is that its validity varies depending on specific sectors or contexts (46, 49). If its generality involves the difficulty of comparing different studies, on the other hand, it has allowed a wave of applications across several fields. While various studies have exploited it in contexts such as the growth patterns of city sizes (62–64), research (65), and human activity (66), its implications for digital domains remain unexamined. Focusing on the supply and demand of news in the attention economy of social media platforms, we aim to determine whether the principles of proportionate growth hold in social media news dissemination.

We systematically study the growth patterns of news outlets on Facebook, comparing their growth to audience sizes over different periods. For a deeper understanding of news engagement on social media, we obtain a list of news outlets from NewsGuard (67), an entity recognized for tackling misinformation by assessing the credibility and reliability of news sources. After selecting all the news outlets with a Facebook account listed on NewsGuard, we use their Facebook URLs to gather their data from CrowdTangle (68), a Facebook-owned tool that monitors interactions on public content from Facebook pages, groups, and verified profiles. This effort provides a comprehensive dataset: the Facebook historical data, from 2008 to the end of 2022, of over 1000 news outlets across four languages—English, French, German, and Italian. Thanks to the post-level granularity of our dataset, we can measure the growth of pages’ metrics on various timescales by aggregating data according to a broader or narrower time window (daily, weekly, monthly, and quarterly), providing robust insights into online news outlets’ growth dynamics.

The article is structured as follows: Initially, we define our analysis framework, investigate the growth regime, and assess its dynamics. Next, we introduce a stochastic model to replicate the observed growth patterns, illustrating the consistency between results and empirical evidence. Finally, we compare the growth of news outlets based on their information quality. We find that the ability to create viral content and capture widespread attention is untied to the size of the information provider. Engagement follows a universal growth pattern in short-term intervals. Contrary to common belief, we observe that the number of Followers is not a reliable measure of a page’s peaks of influence; the impact on engagement becomes apparent only over extended periods. Additionally, we discover that the unreliability of a news source negatively affects engagement growth in the long term.

## Results

We start by defining our framework of analysis. The simplest growth model, proposed by Gibrat (45), states that a given company’s proportional growth rate is independent of its absolute initial size. His assumptions can be formalized by the following random multiplicative process for the size *S*:


(1) $$S_{t+\Delta t}=S_{t}(1+\epsilon_{t}),$$

where t≥0 is time, Δt>0, $S_{t+\Delta t}$, and $S_{t}$ are the sizes at time t+Δt and *t*, respectively, and $\epsilon_{t}$ is a random variable coming from an i.i.d. stochastic process uncorrelated to $S_{s}$ (0≤s≤t) having mean *μ* and standard deviation *σ*. Due to the generic formulation of the original model, we adapt its interpretation to achieve a meaningful application in the context of social media. In terms of information spreading, virality refers to the rapid and widespread dissemination of information or content. By focusing on the extent and impact of the diffusion, virality refers to content engagement exceeding typical expectations, reaching a massive number of users and interactions. Therefore, virality is the widespread diffusion and over-engaging performance of a piece of content in the short term. In our analysis we focus on the latter facet, characterizing the growth of content performance with respect to the size of its source. The analysis is performed on two key metrics: Followers and Engagement. We first define how to assess page size and performance on social media, and our timescales of analysis. Then, we evaluate the growth regime of both metrics concerning size for each timescale.

### Metrics and methodology for social media page analysis

In evaluating whether the size of a page affects its growth, we first need to establish how to measure the size and its performance. In the study of social media platforms, notably Facebook, we primarily rely on two metrics: (i) Page Followers, the number of users subscribed to a given page at the time of posting, representing a metric of reach and (ii) Engagement, encompassing the total number of users’ interactions with the page’s posts (i.e. the sum of Likes, Comments, and Shares). The size of a page is typically inferred from its Followers count (69–72), a standard measure on such platforms. The alternative would be using Engagement, but such a choice would introduce undesired issues. Indeed, the engagement definition is inherently ambiguous: it can be a cumulative sum of interactions over the entire lifespan or a count over a specific duration, such as a week. With period-specific measures, pages’ size could fluctuate too widely (spanning even across orders of magnitude), thus leading to interpretational challenges. Conversely, using a cumulative engagement count to quantify size may over-represent past performances. Consequently, we opt to use Followers as a more stable representation of page size. On the other hand, Engagement represents the page performance in terms of users’ attention.

Our analysis leverages varied timescales to observe growth patterns. Specifically, we consider four time-granularity: daily (D), weekly (W), monthly (M), and quarterly (Q). Therefore, for each page, we consider both metrics, Followers and Engagement, according to different time windows. To measure engagement, we consider aggregated data depending on the timescale of the analysis, thus computing the total engagement instead of its mean value. Focusing on the total attention received by the news outlets, we consider higher total engagement as a higher users’ attention, regardless of the number of posts. Such interpretation relies on the fact that publishing more posts does not lead to more engagement if users are not interested in a topic. Likewise, getting engagement with several posts implies high attention, and using the mean value would underestimate the latter. Apart from these differences, the information captured by total and mean engagement is similar in many cases. We further validate it by showing that both measures bring comparable results in this analysis. For Followers, since they already are a cumulative value, we take only a representative data point in the time window, depending on the chosen timescale (see Materials and methods for further details). Transitioning between these scales offers diverse and new perspectives on growth dynamics. In the case of Followers’ growth, daily measurements are deemed unsuitable due to limited variability, whereas all four scales are relevant for Engagement analysis, since news outlets are very active accounts and usually have multiple posts per day.

### Assessing the growth regime

Based on the definition of Followers and Engagement, and according to (1), we refer to Followers and Engagement growth, respectively, as


(2) $$F_{t+\Delta t}=F_{t}(1+\epsilon_{t}^{\left(F\right)})$$

(3) $$E_{t+\Delta t}=E_{t}(1+\epsilon_{t}^{\left(E\right)}),$$

where the superscripts (F) and (E) point to an intuitive notation for the process ϵ with mean $\mu_{F}$ and $\mu_{E}$ and standard deviation $\sigma_{F}$ and $\sigma_{E}$ for Followers and Engagement, respectively. Time measures the different timescales: D, W, M, and Q. In (2), $F_{t}$ is the number of Followers at time *t* while $E_{t}$ represents the number of interactions generated at time *t*. In the same way, $\left(1+\epsilon_{t}^{\left(F\right)}\right)$ and $\left(1+\epsilon_{t}^{\left(E\right)}\right)$ are the growth rates of $F_{t}$ and $E_{t}$, respectively. As Gibrat’s Law was originally intended to explain the emergence of a log-normal distribution of sizes, we first assess that Followers and Engagement distributions comply with this assumption. Since Followers’ records on CrowdTangle start from 2018 January 1 and stop on 2022 December 31 hence relying on a 5-year timespan of analysis, we take into consideration such a period for the relationship between Followers and Engagement growth. For this reason, when we consider the metrics of Followers and Engagement jointly, we restrict our analysis period to 2018 January 1–2022 December 31. In the analysis in which we do not account for Followers’ value, we consider the entire 15-year timespan, ranging from 2008 January 1 to 2022 December 31. See Processing methods section and Fig. S1 for further details. Figure S2 shows distributions of both metrics at the start and end of the considered period.

To assess whether growth rate distributions vary based on page size, we define four classes of pages based on their Followers, so as to have comparable populations between them over the entire period. The considered four classes of Followers are: 10 K–50 K, 50 K–150 K, 150 K–500 K, and 500 K–5 M. The bin boundaries were defined by jointly considering two aspects: a comparable number of pages between classes and actual values of Followers for which it was reasonable to account for a page as small, medium, large, or very large. As a robustness check, we reported the clustering in Fig. S3 from which our classes and the clustering ones are predominantly overlapping.

**Fig. 1.**
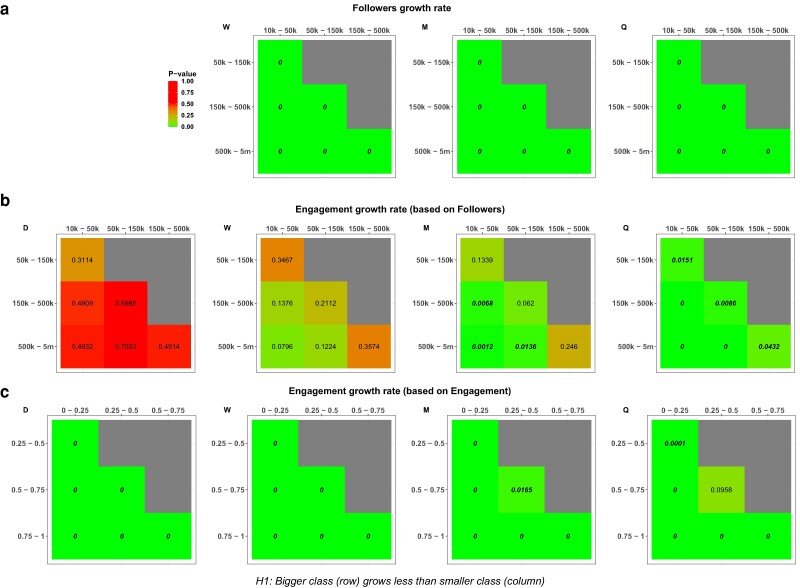
a–c) *P*-values of Mann–Whitney *U* tests between classes of size for Followers and Engagement growth rate distributions. Panel titles indicate the metric being tested and the metric according to which we determine the size. Row and column headers represent the class size. Bold numbers represent *P*-values for which we reject the hypothesis that the growth distributions do not differ, with the alternative hypothesis that the smaller class grows at a higher rate. For readability, 0 represents *P*-values smaller than 0.0001.

In evaluating growth regimes, we posit that the absence of size effects should, as an initial assumption, result in comparable growth rate distributions across different classes. To compare growth rate distributions among different classes of size, we apply, to each pair of bins, a Mann–Whitney *U* test on both metrics and for different timescales. In Fig. 1, we reported the tests with the alternative hypothesis that the smaller class grows at a higher rate, showing significant results, while Fig. S4 reports *P*-values of the two-tailed tests. We first inspect the case of Followers, reported in Fig. 1a. Statistical tests across observed timescales consistently demonstrate that smaller pages experience greater Followers’ growth than their larger counterparts. This evidence counters the notion of proportionate effect growth as described by Gibrat.

In contrast, the growth dynamics of Engagement provide intriguing insights. Specifically, in Fig. 1b, the growth regime is influenced by the duration of the observed timescale. In short-term observations (daily and weekly scales), engagement variation is consistent irrespective of page size. Thus, from a micro-level perspective, engagement adheres to a universal growth regime, independently of page size. However, as we transition to a monthly scale, a deviation in the regime emerges, with smaller-sized classes outperforming their larger counterparts. This deviation becomes definite on a quarterly timescale, underscoring the influence of size on long-term engagement growth. For a comprehensive perspective, we recalibrated our Engagement analysis, reported in Fig. 1c, categorizing size based on Engagement metric. Bins are delineated by the quartiles of Engagement distribution across all pages within a specific timescale, after trimming between the 5th and the 95th percentiles. Our analysis indicates that, in this case, the system predominantly diverges from Gibrat’s law of proportionate effect. Exceptions are noted for middle-sized pages (those within the 2nd and 3rd quartiles) on a quarterly timescale. Thus, in short-term observations, Engagement consistently depends on its recent performance, irrespective of page size, while the influence of Followers becomes evident with the increase of the observed timescale.

We note that our results show correspondences with evidence from prior studies in different domains, such as the distribution of growth rates displaying a “universal” form that does not depend on the size (65), and the system experiencing a growth regime transition as the timescale widens (73). These findings bear significant implications. Notably, in the short term, size does not dictate the probability of engagement growth. Extrapolating this to individual posts suggests an egalitarian landscape where every news item, irrespective of its source or the number of its Followers, has an equal propensity to go viral, that is to suddenly gain disproportionate engagement. Consequently, the mere count of Followers proves inadequate in gauging the page’s potential influence. As a robustness check, in Figs. S5 and S6, we reported two variants of the tests performed in Fig. 1b, showing how the results still hold by using the mean engagement value or by changing the bin boundaries. Moreover, we further evaluated the relationship between Engagement growth and Followers value, using the latter as a continuous variable by performing a linear regression as


(4) $$\epsilon_{t}^{\left(E\right)}=\beta_{0}+\beta_{1}ln(F_{t}),$$

where $\epsilon_{t}^{\left(E\right)}$ is the logarithm of the Engagement growth rate and $ln(F_{t})$ is the logarithm of the Followers value at time *t*, and by calculating the Pearson Correlation Coefficient between the two across timescales. A graphical representation of the regression results is reported in Fig. S7, while their coefficients are reported in Table S1. Anew, the results are consistent with the overall finding, elucidating how, on short-term scales, the Engagement growth is not affected by size effects, while the latter start to manifest in the long term, with bigger pages showing, on average, a negative growth.

### Analyzing engagement and followers dynamics

**Fig. 2.**
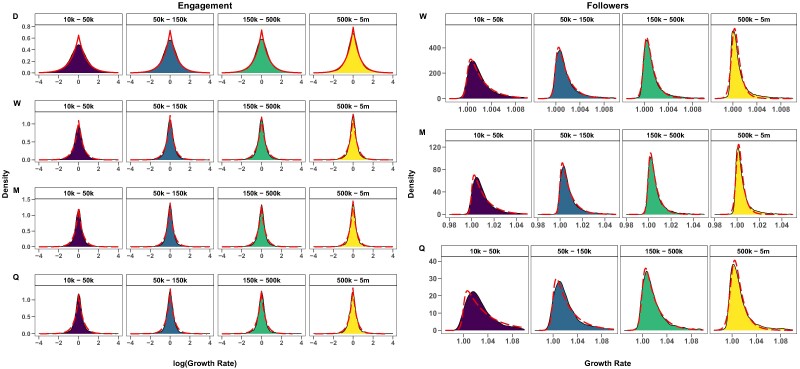
Comparison of observed and theoretical growth rate distributions for Engagement and Followers. Dotted lines denote theoretical densities obtained by fitting empirical ones, with labels D, W, M, and Q indicating Daily, Weekly, Monthly, and Quarterly timescales.

Empirical evidence suggests that the logarithm of many growth rate distributions often takes an exponential form. Consistently with prior studies (55, 74), our analysis reveals that the logarithm of Engagement growth rates adheres to a particular exponential distribution known as the Laplace distribution. In contrast, the growth rates for Followers display an asymmetry, exhibiting a right-skewed distribution. Our analysis suggests a fit with a heavy-tailed distribution, specifically the Burr distribution (75). Since the Burr is exclusively defined for positive values, we here employ the absolute growth rates, rather than their logarithms. A visual comparison between the observed and fitted distributions is reported in Fig. 2 (see Materials and methods section for details of the fitting procedure).

The matching of the empirical distributions of Engagement growth with the Laplace brings significant upshots. As pointed out by previous works (51, 76), growth phenomena could display a nontrivial relation between the positive and negative side of its rate distribution. The detailed balance property, or time-reversal symmetry, states that the empirical probability of changing size from one value to another is statistically the same as that for its reverse process. Statistical tests provided evidence of how the Engagement’s short-term fluctuations adhere to a universal distribution, independently of the page size, with $\mu_{E}\to 0$ when passing from timescale Q to timescale D. Figure S8 shows parameters variation according to timescales for the considered size classes. Furthermore, the symmetry property of the Laplace distribution, with μ≈0, directly implies the validity of detailed balance.

We can draw two significant implications from this outcome. These findings show interesting results: while the extension of the followers of a page obeys a logistic trend-based law, in contrast with Gibrat’s Law—hence, smaller pages grow faster than their bigger counterparts until reaching an equilibrium audience—an inherent fluctuating dynamics underlies the attention of their user bases, unveiling the bounds of collective attention. Assessing these statistical properties of short-term engagement provides a deeper understanding of news consumption dynamics, which can influence how news providers act to handle the information market, in which they compete with each other to capture a fluctuating and scarce resource as users’ attention. From a technical standpoint, ascertaining the universality of this dynamic and the probability distribution that describes it enables us to exploit it as a proxy for defining and detecting virality, namely gaining disproportionate engagement.

### Modeling growth

Our empirical findings show that the impact of size on the Engagement growth becomes evident only if observed through larger timescales and that its growth pattern is universal at the micro-level. Knowing the distributions that define the evolution of our metrics allows us to evaluate the variation of their parameters according to size and timescale. For each time scale, we can model parameters of both growth rate distributions based on Followers and Engagement values. The regression coefficients are reported in Table 1. We can thereby simulate growth on the chosen timescale, given two starting values of Followers and Engagement, $F_{0}$ and $E_{0}$, by iteratively sampling growth rates value from the distributions modeled using the parameters specified in Cases 1 and 2.

**Table 1.** Regression coefficients and *P*-values of Laplace and Burr distribution parameters estimation for different timescales.

β0		β1		β2		Par	Time
− 0.109	(0.063)	0.054	(<0.001)	− 0.062	(<0.001)	*μ*	W
0.073	(0.248)	0.037	(<0.001)	− 0.051	(<0.001)	*μ*	M
0.384	(<0.001)	0.031	(<0.001)	− 0.065	(<0.001)	*μ*	Q
0.613	(<0.001)	0.027	(0.001)	− 0.054	(<0.001)	*b*	W
0.593	(<0.001)	0.041	(<0.001)	− 0.066	(<0.001)	*b*	M
0.844	(<0.001)	0.056	(<0.001)	− 0.094	(<0.001)	*b*	Q
8,420.469	(<0.001)	− 372.77	(0.025)	–		*c*	W
2,550.01	(<0.001)	− 127.559	(0.014)	–		*c*	M
1,053.905	(0.002)	− 56.113	(0.017)	–		*c*	Q
− 0.778	(<0.001)	0.083	(<0.001)	–		*k*	W
− 0.751	(<0.001)	0.078	(<0.001)	–		*k*	M
− 0.714	(0.001)	0.073	(<0.001)	–		*k*	Q

#### Case 1: Engagement growth

We consider the dynamics described in (3) with $\epsilon^{\left(E\right)}$ following the Laplace distribution in (9) (see Materials and methods), with:


(5) $$\mu =\beta_{0\mu }+\beta_{1\mu }ln(F_{t})+\beta_{2\mu }ln(E_{t})$$

(6) $$b=\beta_{0b}+\beta_{1b}ln(F_{t})+\beta_{2b}ln(E_{t})$$

#### Case 2: Followers growth

We consider (2), being $\epsilon^{\left(F\right)}$ the logarithm of the growth rate behaving according to the Burr distribution in (11) (see Materials and methods), with:


(7) $$c=\beta_{0c}+\beta_{1c}ln(F_{t})$$

(8) $$k=\beta_{0k}+\beta_{1k}ln(F_{t})$$

**Fig. 3.**
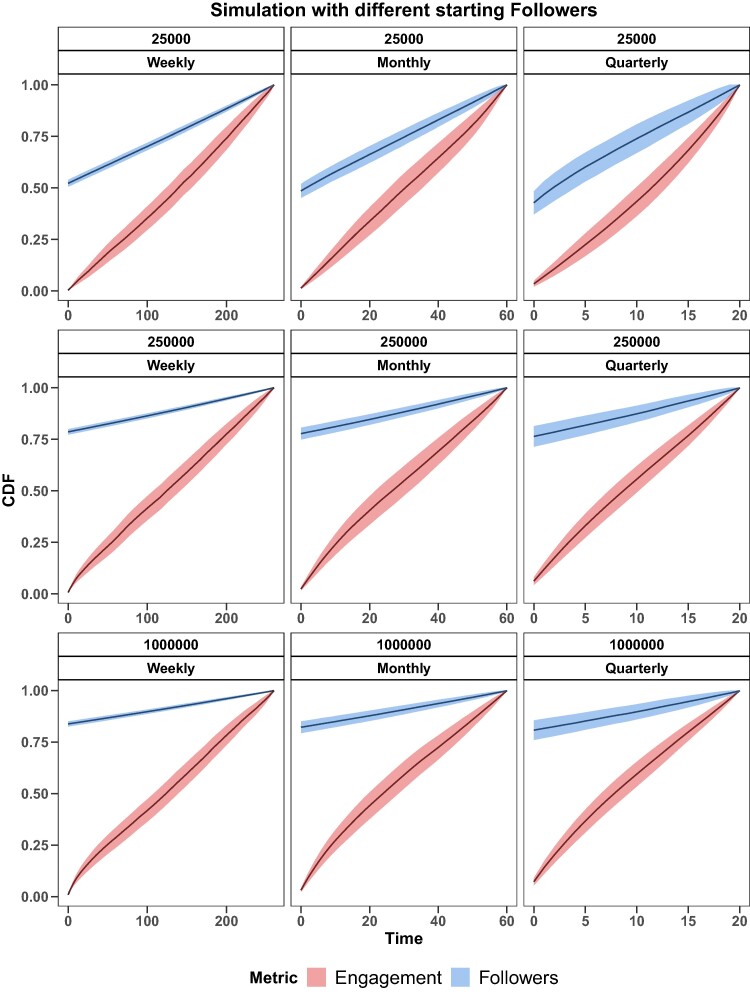
Results of growth simulation with different starting sizes. Sub-plot headers indicate the Followers starting value and the related timescale. Solid lines represent the mean cumulative distribution function value of the iteration time, shades represent the corresponding standard error.

Results of simulations are shown in Fig. 3, representing the evolution of both metrics for different starting sizes (Followers) on three timescales used for the analysis (W, M, and Q). We selected three starting sizes representing pages with low, medium, and high number of Followers, i.e. 25K, 250K, and 1M, respectively.

As results show, by observing the system on a weekly timescale, the engagement shows a basically steady evolution for all three sizes. As we extend the observed timescale, by passing from weekly to quarterly, the engagement growth of small pages begins to exhibit convex behavior, while the growth curve of big pages shifts toward concavity, providing evidence of how Followers impact the engagement evolution only over long-term intervals. On the other hand, Followers of smaller pages always grow faster than bigger ones in each timescale. Our simulations consistently match empirical evidences. Despite the model’s engagement growth probability being based on Followers, the universal characteristic of the process at the temporal micro-scale level is evident.

These results highlight the limitation of using Followers as the sole metric to gauge overall page influence. Short-term outcomes seem to derive from a uniform stochastic process, possibly elucidating the influence of algorithms on user news consumption behaviors. While this suggests an environment where all content providers might be on an equal stand regarding visibility, it also necessitates continuous monitoring to mitigate the spread of harmful content, such as misinformation. Basing influence assessments solely on the number of Followers can lead to oversight. The potential presence of “one-time” or “hidden influencers”—entities with a disproportionate influence relative to their Follower count—needs attention. The missing of a clear engagement effect on Follower growth, the lack of significance of β2 on Burr’s parameters variation, further emphasizes this, indicating that heightened interactions do not necessarily translate to a corresponding increase in Followers or sustained reach.

### Growth and information quality

**Fig. 4.**
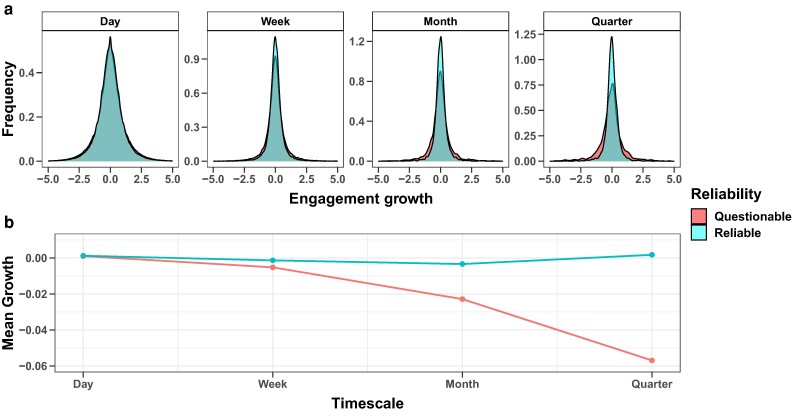
a) Comparison of Engagement growth rate distributions of Questionable and Reliable pages for different timescales. b) Mean growths of Questionable and Reliable pages across increasing timescales.

The effects of external factors on engagement growth manifest in the long term. Potential explanations for differences in page growth could be plenty, though one of the most relevant for society is the propensity of news outlets to produce unverified news and misinformation. For this reason, we conclude our analysis by comparing two sub-samples of pages representing reliable and questionable ones. Since we do not account for Followers value, this analysis encompasses the entire pages’ lifespan. The classification is performed based on reliability scores provided by Newsguard. Since our dataset comprises 898 reliable sources and 131 nonreliable ones, we performed a sampling of 131 reliable sources to obtain two comparable samples with similar structural characteristics, namely Followers and Page’s lifespan. We selected the partition of 131 Reliable pages for which, using Followers and Lifespan as distance variables in a 2D space, the sum of their Euclidean distances from the 131 Questionable pages was minimized. See Materials and methods and Fig. S8 for further details about the reliability ratings and the sampling procedure. Figure 4 shows growth rate distributions of engagement and their evolution across the various timescales. Table S2 shows the *P*-values of Mann–Whitney *U* tests between the two sub-samples, as in our previous analyses, and Table S3 shows the same tests using the entire set of Reliable pages. Both graphic representations and tests display how the trustworthiness of the news source plays a crucial role, as the engagement of unreliable pages progressively decreases as the time scale widens. Anew, the short-term fluctuations follow a universal dynamic, and neither the reliability turns out to determine growth differences. For a comprehensive perspective, we also tested the Followers’ growth based on the trustworthiness of the pages. A graphical representation is reported in Fig. S10, while Table S4 reports the *P*-values of Mann–Whitney *U* tests. Again, Reliable pages show a greater growth in each timescale, with a noteworthy peculiarity. As Panels A and B of Fig. S10 show, the distributions of Reliable pages are distinctly right-shifted compared to the Questionable ones, as confirmed by the results in Table S4. However, Questionable distributions simultaneously show a lower peak of frequency and a heavier tail of growth rates. This evidence sheds light on the turbulent dynamic of the audience of untrustworthy sources, which regularly exhibit a very low—at times negative—growth, with bursts of striking sudden increases. The long-term divergence, which may result from both users’ behavior and platform moderation policies, along with the inherent randomness of short-term fluctuations, highlight the importance of continuous efforts to monitor the production, diffusion, and consumption of sensitive content, such as misinformation, unsubstantiated content, and nonrigorous journalism. The effects that we note may also be explained by several other factors. For instance, confirmation bias and selective exposure are among the main drivers of how people build their connections, select sources and information, and spread them (18, 77). Furthermore, engagement may be affected by the enforcement of moderation policies aimed at reducing the diffusion of extreme content that could nonetheless lead to the rise of partisan and radicalized communities (78, 79). Therefore, being aware of factors such as the structure of the diffusion network (80, 81), the rise and fall of support of specific topics (82), and other forms of users behavior (83, 84) could be relevant to take into account potential confounding factors on the evolution of engagement.

### Conclusions

In historical media landscapes, prominent news outlets predominantly influenced agenda-setting (7, 9), their reach determining the flow and focus of public discourse. However, the emergence of social media platforms—designed more for entertainment than information spreading—has reshaped this dynamic. While many assume that larger news outlets and their inherent reach would still dominate the discourse in the social media environment, our research challenges this perspective. Analyzing engagement metrics across diverse news outlets on Facebook, we find that news virality, namely a sudden and disproportionate growth of content’s engagement in the short term, is not strictly tied to the traditional size of the outlet. Instead, a myriad of factors may drive online discourse: the rapid increase of the engagement of a topic (85), the reinforcing nature of echo chambers (24), the amplifying power of influencers (13, 69), the emotional resonance of content (6, 20), and even artificial amplification via bots (86). This complex web of drivers, some of which seems to exhibit random behaviors, defies conventional models of media influence. Indeed, understanding the dynamics of the attention economy is pivotal for charting the trajectory of content creators on platforms like Facebook.

In this work, we analyze a massive dataset composed of 57 million posts comprising the entire Facebook history, spanning 15 years, of over 1,000 news outlets. In particular, this study took a deep dive into these dynamics, evaluating the applicability of Gibrat’s Law—a principle traditionally applied to business growth—in social media content creation. Empirical results provided a nuanced understanding of growth patterns. We observe that the likelihood of generating viral content and capturing widespread attention is independent of the information provider’s size. Indeed, engagement adheres to a universal growth pattern in short-term intervals. This pattern shifts as the analysis extends to longer timescales like monthly and quarterly intervals, where size effects begin to manifest. We validated this dynamic by comparing news outlets’ growth based on their information quality, providing evidence on how, though the unreliability of the news source negatively impacts engagement growth, its effect only manifests in the long term. Another significant observation challenges conventional wisdom: page’s Followers are not a sufficient indicator of the potential influence of a piece of content in the near term, and their actual impact only emerges over extended periods. Our examination of growth dynamics further elucidated these insights. After detecting their probability distributions, we evaluated their behavior according to size and timescale. We developed a stochastic model validating our empirical findings, emphasizing that Followers do not always depict actual influence or engagement potential in the short term.

This brings broader implications in the context of agenda-setting dynamics in the social media era. Our study shows that contrary to traditional media, influence is not strictly related to size or following in the digital realm. With the short-term evolution of engagement being untied to the number of Followers, these results support the hypothesis that the engagement is not a matter of the source but is rather more likely related to the content. This stochastic nature of short-term engagement suggests an environment where all content, irrespective of its source, stands a roughly equal chance of capturing attention, possibly elucidating the influence of algorithms on users’ news consumption. Focusing on the prioritization criteria of the recommendation algorithm, a higher emphasis on the content quality rather than the source would certainly represent a more egalitarian scenario of news dissemination and consumption. This democratization of potential attention influences how narratives and agendas are set, with even smaller entities having the power to shape discourse. However, it also emphasizes the importance of vigilant monitoring mechanisms, given the risk of rapid misinformation or harmful content spread. Our research highlights the intricate dynamics of growth in the digital attention economy, revealing how traditional metrics may not align with real-world influence. It also offers key insights into how the modern agenda-setting dynamics are being reshaped in the era of social media. These findings are precious for content creators, platform designers, and policymakers as they navigate the complexities of the digital age.

The work presents some limitations in data availability and the generalization of the dynamics to other platforms. Regarding the first, since Followers’ data are accessible starting from 2018 January 1, it was not possible to evaluate the size dynamics since the platform’s early years. Since Facebook is a long-standing and established platform, future research could investigate such dynamics in newer or less developed platforms. Regarding the second, the different growth patterns of page size could differ between social media platforms and their life stages. Future research could evaluate whether the engagement patterns, consistent with other human dynamics observed in various domains, generally hold in different social media platforms. Furthermore, having available sufficient data about content features would make it possible to investigate whether and which media characteristics drive the reach and the engagement of news on social media.

## Materials and methods

### Selecting followers’ value

Since the pages are news outlets, they usually have at least one post per day and consequently we can track the number of Followers at the time of posting. However, not all of them may have posted every single day. To overcome this issue, in determining the Followers’ value for each time window, we selected the closest observation to a given time point of the window, which we referred to as a “representative point in time” since it varies depending on the timescale. For the weekly scale, we selected the value on the minimum observed date of the week. For the monthly scale, we selected the value of the closest observed date to the central point of the month (the 15th day). For the quarterly scale, we selected the value of the farther observed date. The points of reference are selected in such a way as to measure the Followers’ growth at the start, in the middle, and at the end of the respective time window, avoiding multiple selections of the same measurements.

### Labeling of media sources

The reliability labeling of news outlets is based on the trust ratings provided by Newsguard (67). Each site is rated using nine basic, apolitical criteria of journalistic practice, related to credibility and transparency. Based on the nine criteria, each site gets a trust score of 0–100 points. NewsGuard labels the source as Trustable if the resulting score equals or exceeds 60. The total number of news outlets for which we have a trust rating is 1,029.

### Parameters estimation

Here, we provide details about the fitting procedure of distributions reported in Fig. 2 of sections Analyzing Engagement and Followers Dynamics, and Modeling Growth.

#### 1. Laplace distribution

The probability density function of the Engagement growth rate is the Laplace distribution, expressed as:


(9) $$f_{L}(x|\mu ,b)=\frac{1}{2b}\exp\;\left(-\frac{\left|x-\mu \right|}{b}\right),$$

where x∈R and *μ* and *b* are parameters to be calibrated. In this respect, the parameters of the Laplace distribution can be derived analytically from the mean $\mu_{X}$ and standard deviation $\sigma_{X}$ of the empirical distribution *X*, since


(10) $$\mu =\mu_{X};\;b=\frac{\sigma_{X}}{\sqrt{2}}$$

#### 2. Burr distribution

The probability density function for Followers’ growth is described by the Burr distribution, whose density is:


(11) $$f_{B}(x|c,k)=ck\frac{x^{c-1}}{(1+x^{c}{)}^{k+1}},$$

where x∈R and *c* and *k* are scalars to be calibrated. In particular, such parameters are evaluated by fitting the empirical cumulative distribution function of the observed growth rates with the Burr’s one.

#### Regression of distribution parameters

To model parameters variation according to Followers and Engagement values, we first applied the fitting procedure described above to the growth distributions of the sub-samples obtained by binning based on Followers and Engagement, after trimming within the 5th and 95th percentiles of our observed distributions, in each timescale. After obtaining the parameters of the Laplace and Burr distribution of each sub-sample, we performed the parameter regression as described by Eqs. 5, 6, 7, and 8, of section Modeling growth.

### Sampling of reliable news outlets

According to NewsGuard ratings, our dataset comprises 898 reliable sources and 131 nonreliable ones. We performed a sampling of 131 reliable sources to have two comparable samples. To obtain similar structural characteristics, namely Followers and Page’s lifespan, the distance is computed using the maximum observed number of Followers and the page’s creation date as distance variables, since most pages’ last observations coincide with the end of the analyzed period. The resulting sample is obtained by computing the Euclidean distances of all the possible couples of Questionable and Reliable pages in a 2D space, using Followers and Lifespan as space variables. Then, we selected the partition of 131 Reliable pages for which the sum of their Euclidean distances from the 131 Questionable pages was minimized.

## Supplementary Material

pgae257_Supplementary_Data

## Data Availability

The data collection and analysis process are compliant with the terms and conditions imposed by Crowdtangle (68). Therefore, the results described in this article cannot be exploited to infer the identity of the accounts involved. CrowdTangle does not include paid ads unless those ads began as organic, nonpaid posts that were subsequently “boosted” using Facebook’s advertising tools. It also does not include activity on private accounts, or posts made visible only to specific groups of followers. We provide the list of pages we use in our analysis at the bottom of the Supplementary Appendix. The table reports the page name, its Facebook URL, the language of its contents, and the reliability label for the pages we select in the analysis of Growth and information quality section, by which the latter can be replicated. For replicating all other sections, it is sufficient to have the data of the pages downloadable through CrowdTangle by using their URLs and following our procedure explained in Data collection section in the Supplementary Appendix.

## Preprints

A preprint of this article is published at https://arxiv.org/abs/2401.17890.
