# Supplementary material for: Followers do not dictate the virality of news outlets on social media
Source: PNAS Nexus. 2024 Jun 28;3(7):pgae257. doi: 10.1093/pnasnexus/pgae257 (PMC11235336; doi:10.1093/pnasnexus/pgae257)
Supplement: pgae257_Supplementary_Data [file pgae257_supplementary_data.pdf]

Supporting Information for

Followers do not dictate the virality of news outlets on social media

Emanuele Sangiorgio, Matteo Cinelli, Roy Cerqueti, Walter Quattrociocchi

Emanuele Sangiorgio.
E-mail: emanuele.sangiorgio@uniroma1.it

This PDF file includes:

Supporting text
Figs. S1 to S10
Tables S1 to S5
SI References

Supporting Information Text

1. Data Collection

We download our data from CrowdTangle, a Facebook-owned tool that monitors interactions on public content from Facebook pages, groups, and verified profiles. CrowdTangle is accessible to researchers upon request at this link https://www.crowdtangle.com/request. We obtain the list of news outlets employed in the analysis via NewsGuard, which provides, for each outlet, more than 30 distinct categories of descriptive metadata, including a breakdown of their assessment, an indication of its political slant, descriptions of the topics—or types of misinformation—it covers, and more. After selecting all the news outlets with a Facebook account listed on NewsGuard, we use their Facebook URLs to gather their data on CrowdTangle. Using the tool 'Historical Data' provided by CrowdTangle, we download the entire history of each page from its creation date as a table containing information regarding each posted item in chronological order.

2. Processing Methods

With a post-level granularity, the table's columns include several relevant fields, including the post type (link, image, video), its text, the number of reactions to the post, and more. Among these columns, we select two relevant ones: *Total Interactions* and *Followers at Posting*. The Total Interactions column contains the total number of reactions per post (that is, the sum of Likes, Comments, and Shares) that we aggregate (sum) depending on the time scale of the analysis. The Total Interactions are thus our metric accounting for the Engagement. These data are available for the whole page history, that is, for the longest-running pages, from the beginning of 2008 to the end of 2022, when we downloaded data. Since pages' creation dates span through time, our analysis of growth rates is independent of the creation date once accounted for a sufficient life span and activity of the page. The cumulative time series regarding existing pages starting to be active on Facebook is reported in panel A of Fig. S1. The number of pages included in the dataset is 1082, and 94 % of them were created before 01/01/2018. The second variable we use for our analysis is Followers at Posting, which represents the number of users subscribed to a given page at the time of posting. This information, however, is only available for posts made since 01/01/2018. Before that day, CrowdTangle was not collecting such information, or it is not sharing it with end users as of now. For this reason, when we consider the metrics of Followers and Engagement jointly, we restrict our analysis period to 01/01/2018 - 31/12/2022. In the analysis in which we do not account for Followers' value, we consider the entire 15-year timespan. Quantitatively speaking, in panel B of Fig. S1, we report the evolution of the number of posts we consider in the analysis, which is around 57 million, and the Total Interactions, which is around 21 billion, over time. We note from 1/1/2018 onward, 63 % of posts and 56 % of Total Interactions were produced.

3. Data

We provide the list of pages we use in our analysis at the bottom of the SI Appendix. The table reports the page name, its Facebook URL, the language of its contents, and the reliability label for the pages we select in the analysis of Section Growth and Information Quality, by which the latter can be replicated. For replicating all other Sections, it is sufficient to have the data of the pages downloadable through CrowdTangle by using their URLs and following our procedure explained in Section Data Collection in the SI Appendix.

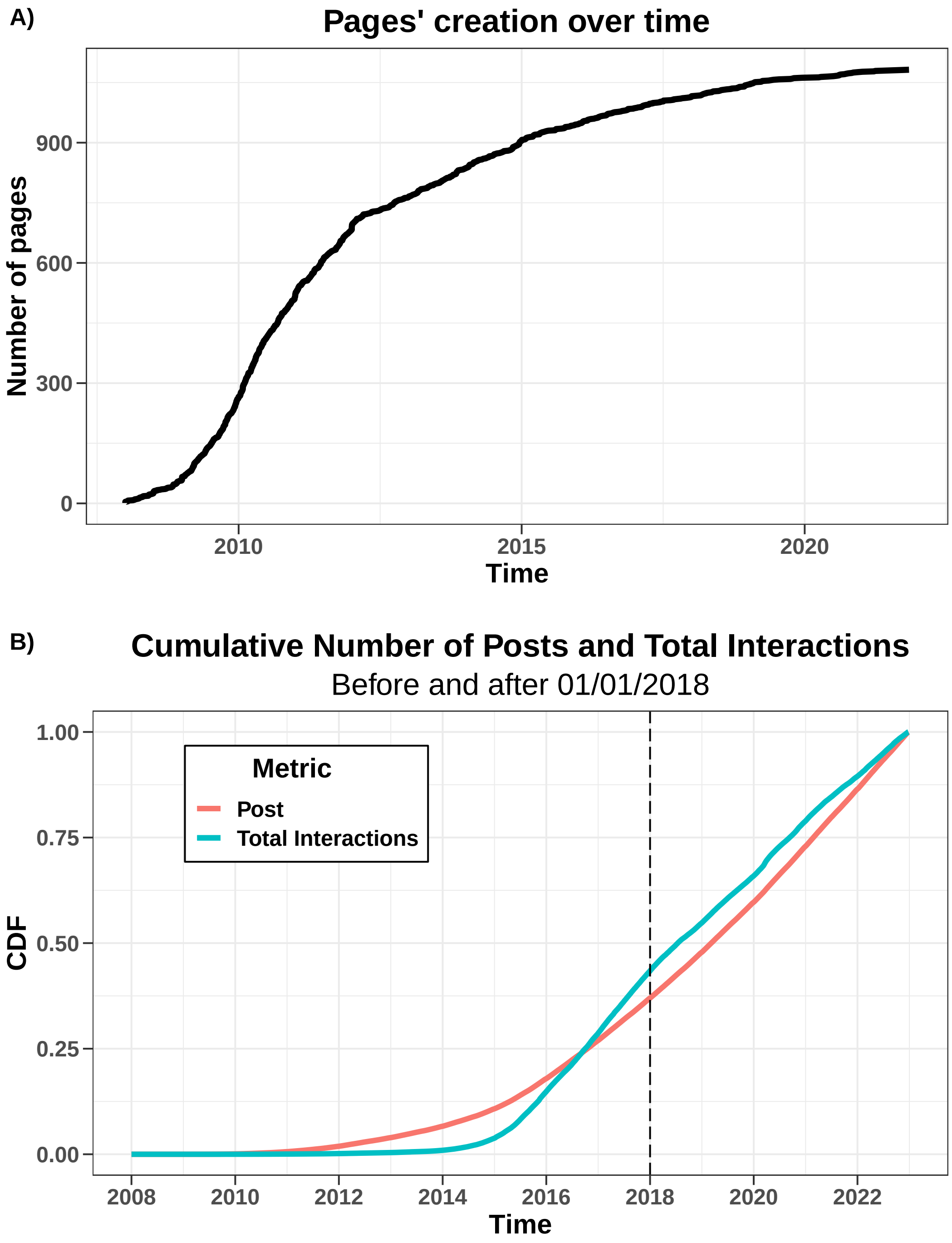

Fig. S1. (A) Pages' creation across time. (B) Evolution of the number of posts and Total Interactions over time.

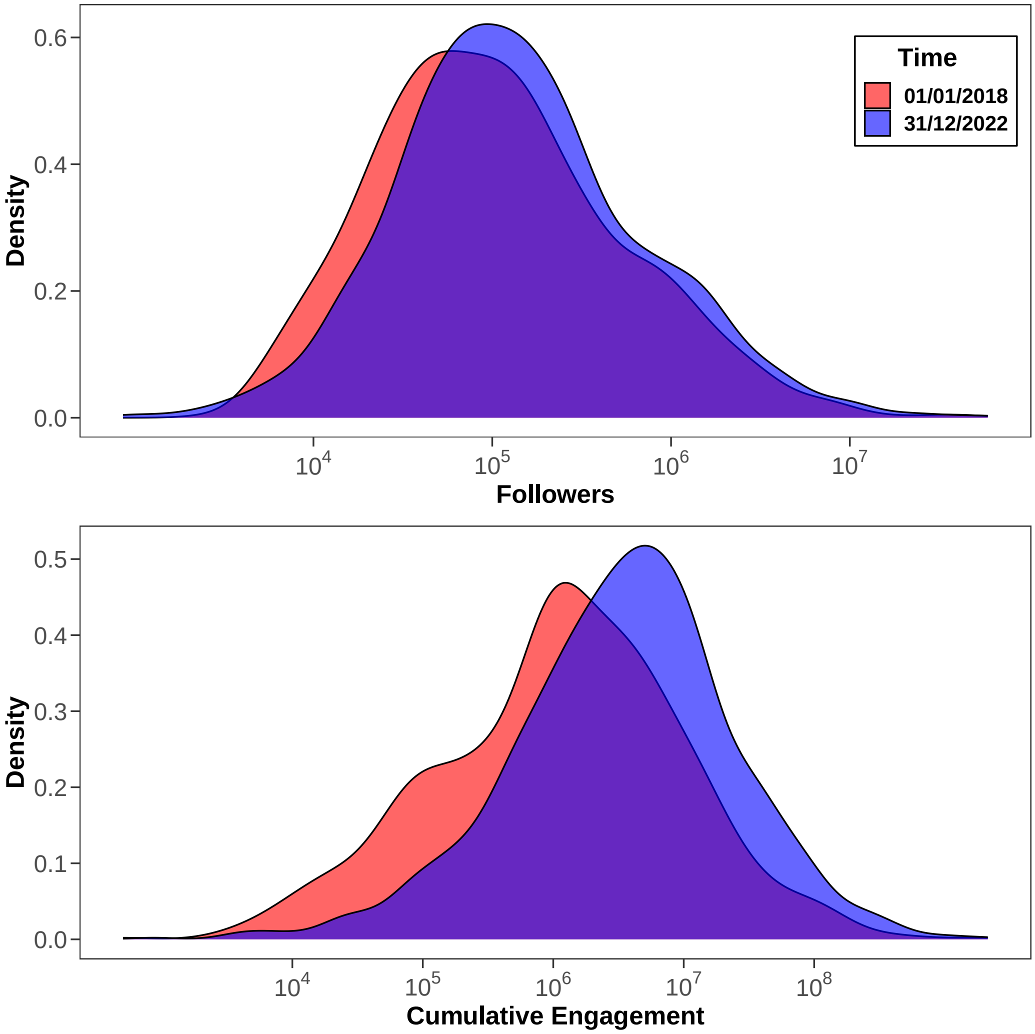

Fig. S2. Distributions of both possible size indicators, Followers and Cumulative Engagement, of the entire sample at the start and end of our analysis period. Both distributions manifest as heavy-tailed, here displayed on a logarithmic scale.

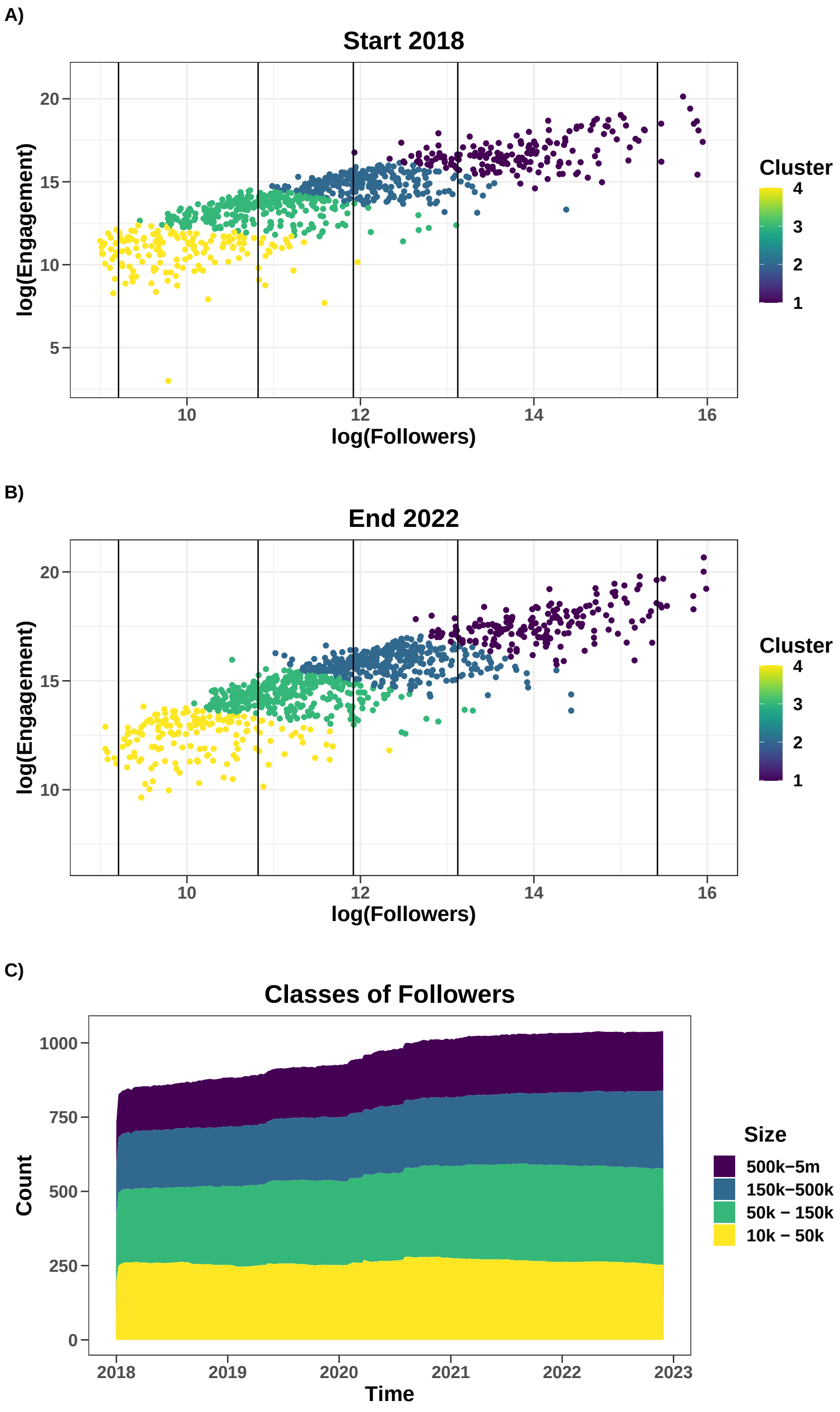

Fig. S3. (A-B) Partitions of PAM clustering of news outlets, based on Followers and Cumulative Engagement values at the start (1/1/2018) and the end (31/12/2022) of our analysis period, respectively. Solid vertical lines represent the range limits of our selected classes of Followers. (C) Frequencies of the size classes across the analysis period.

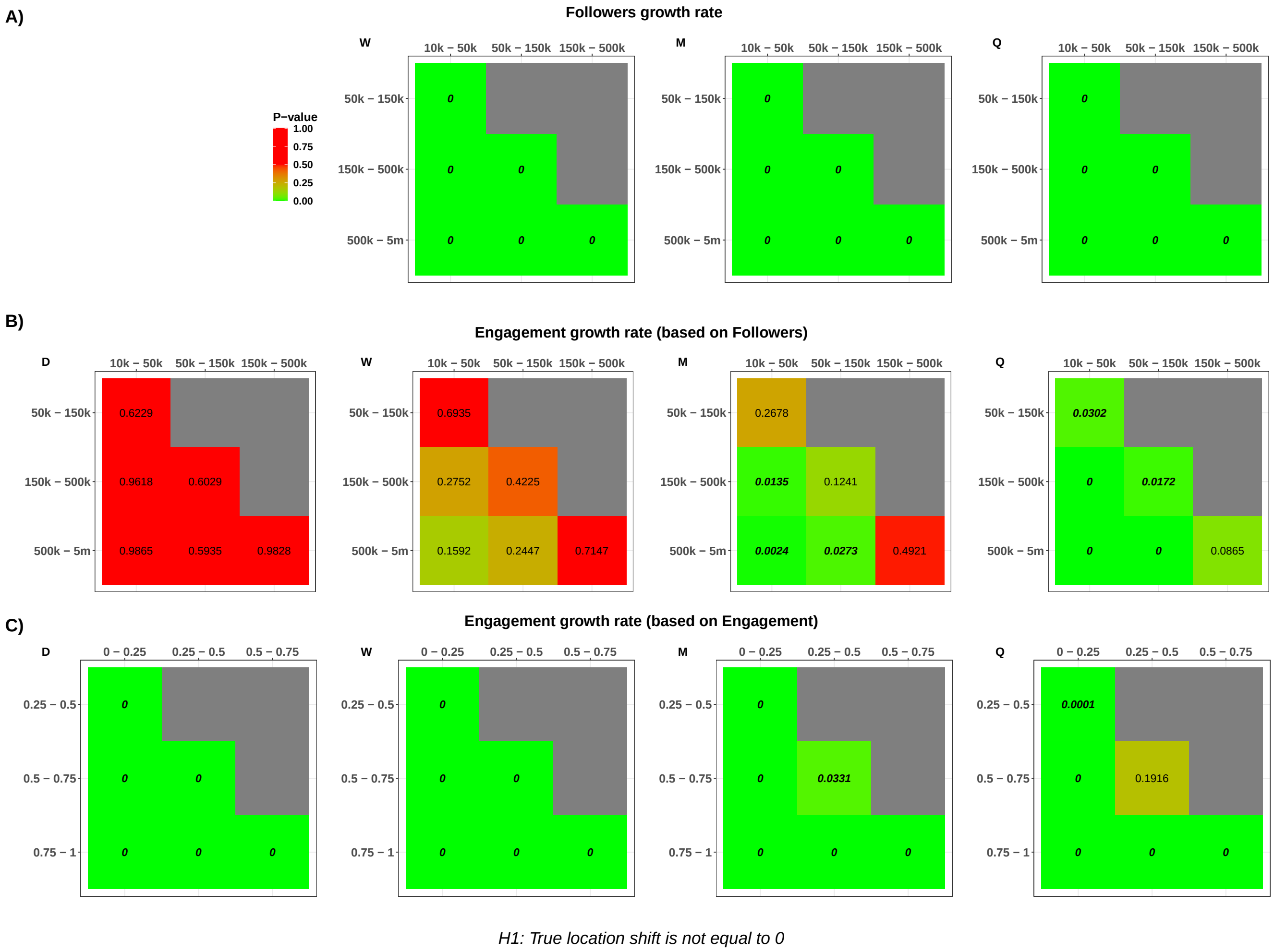

Fig. S4. (A-C) p-values of two-sided Mann-Whitney U tests between classes of size for Followers and Engagement growth rate distributions. Panel titles indicate the metric being tested and the metric according to which we determine the size. Row and column headers represent the class size. Bold numbers represent p-values for which we reject the hypothesis that the growth distributions do not differ, with the alternative hypothesis that the true location shift is not equal to 0. For readability, 0 represents p-values smaller than 0.0001.

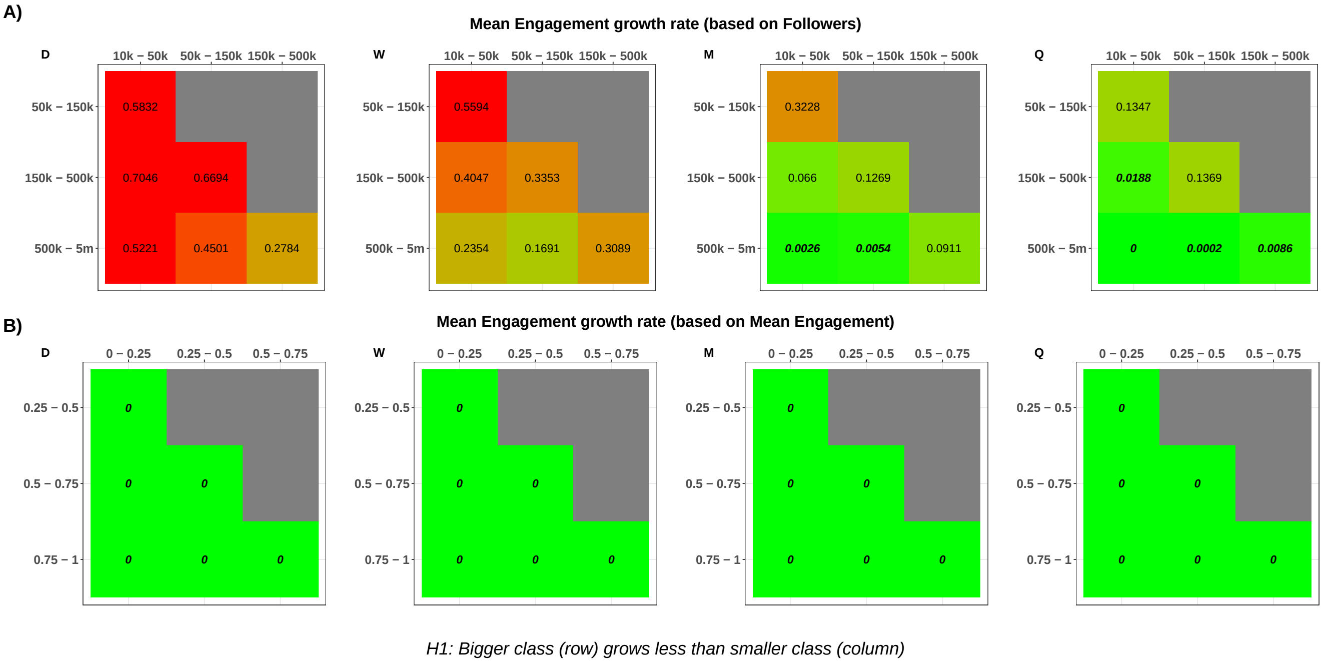

Fig. S5. We recalculated the Engagement using its mean value and repeated the tests reported in Fig. 1B and 1C. (A-C) p-values of two-sided Mann-Whitney U tests between classes of size for Followers and Engagement growth rate distributions. Panel titles indicate the metric being tested and the metric according to which we determine the size. Row and column headers represent the class size. Bold numbers represent p-values for which we reject the hypothesis that the growth distributions do not differ, with the alternative hypothesis that the smaller class grows at a higher rate. For readability, 0 represents p-values smaller than 0.0001.

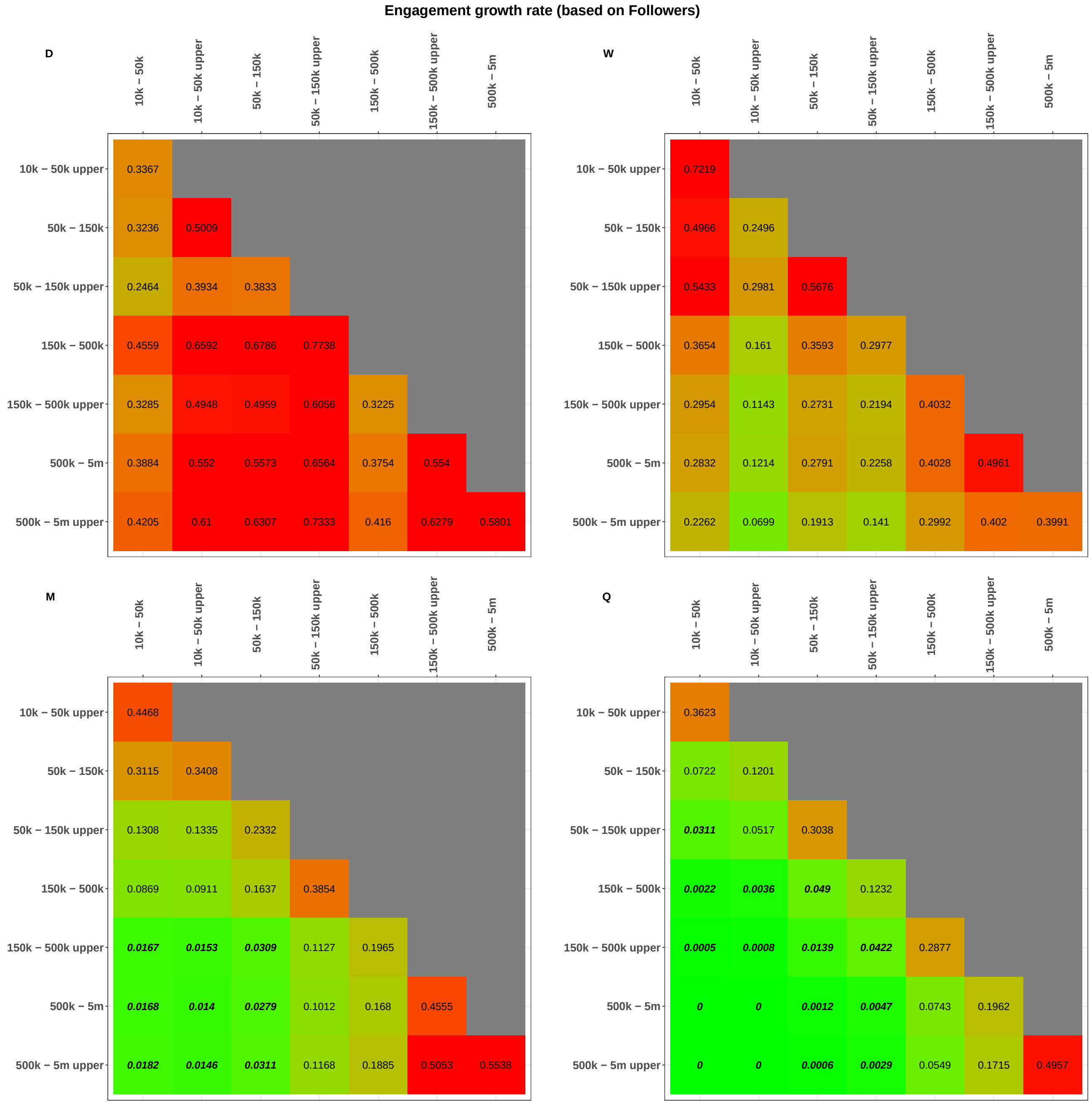

Fig. S6. As the growth dynamic emerges clearly across the different timescales, changing the bin boundaries should not lead to changes in the results. We repeated the tests reported in Fig. 1B and 1C, dividing each original bin into two sub-classes of an equal number of observations by cutting the bin through its Median Followers Value. The independence of growth from size in the short term still holds, with none of the 28 tests showing differences in growth in both Daily and Weekly measurements. On the monthly scale, the three smaller classes grow faster than the three bigger ones, while on the quarterly scale, most tests show statistically significant p-values, except for the pairs of adjacent classes. (A-C) p-values of two-sided Mann-Whitney U tests between classes of size for Followers and Engagement growth rate distributions. Panel titles indicate the metric being tested and the metric according to which we determine the size. Row and column headers represent the class size. Bold numbers represent p-values for which we reject the hypothesis that the growth distributions do not differ, with the alternative hypothesis that the smaller class grows at a higher rate. For readability, 0 represents p-values smaller than 0.0001.

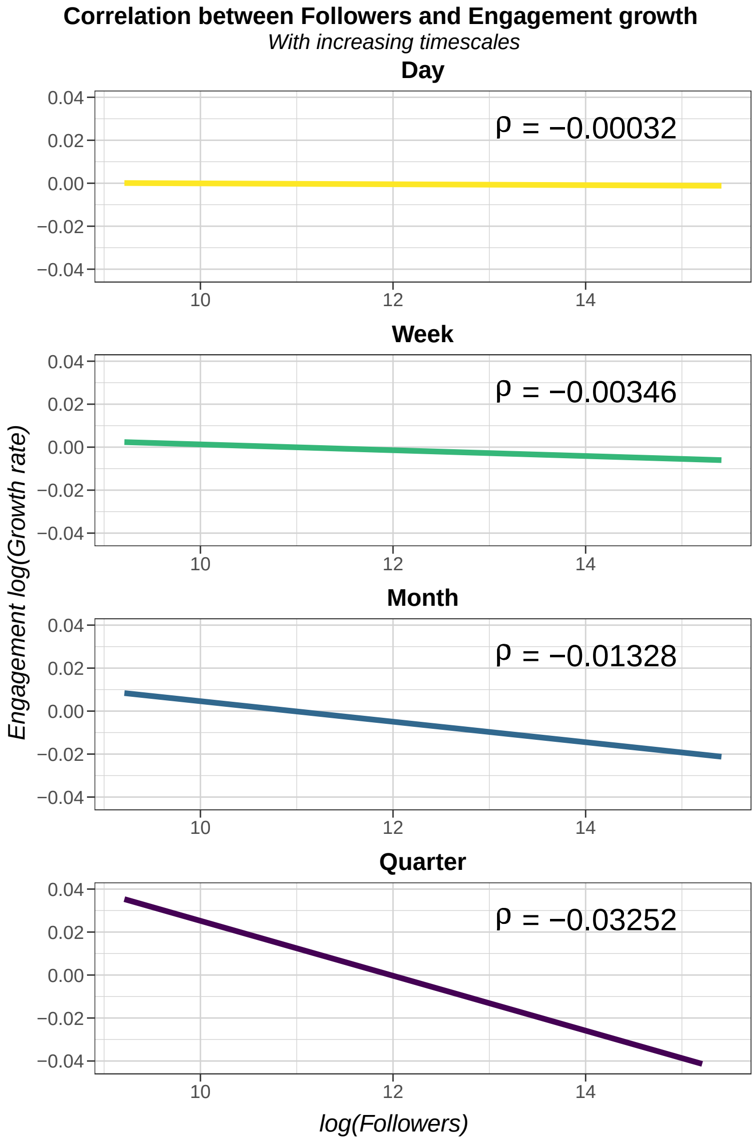

Fig. S7. Correlation between Followers value and Engagement growth on increasing timescales. Lines represent the logarithm of the estimated Engagement growth rate, resulting from the regression coefficients reported in Tab. S1.

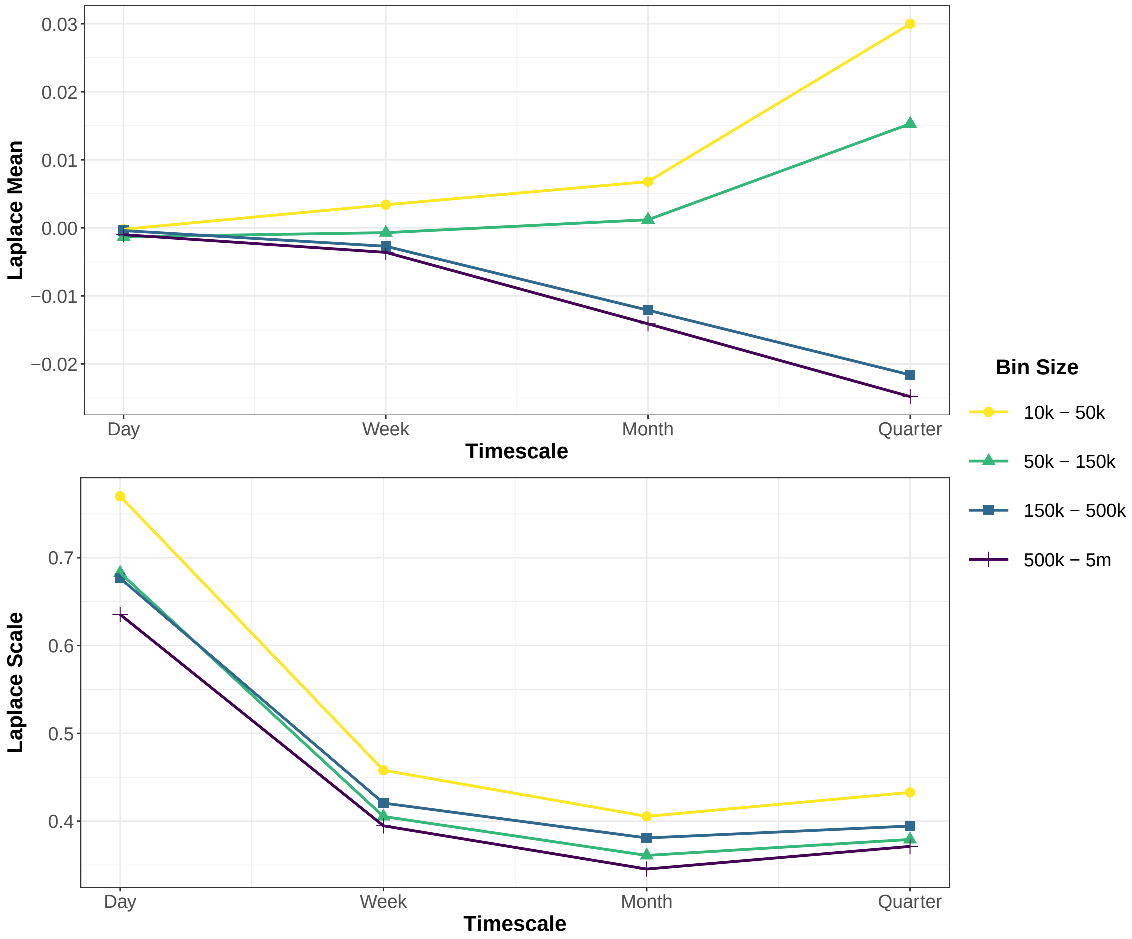

Fig. S8. Laplace parameter variation with the increase of the observed timescale for the four size classes. Upper panel represent μ parameter variation, lower panel represent b parameter variation. As upper panel shows, $\mu \to 0$ with the narrowing of the observed timescale.

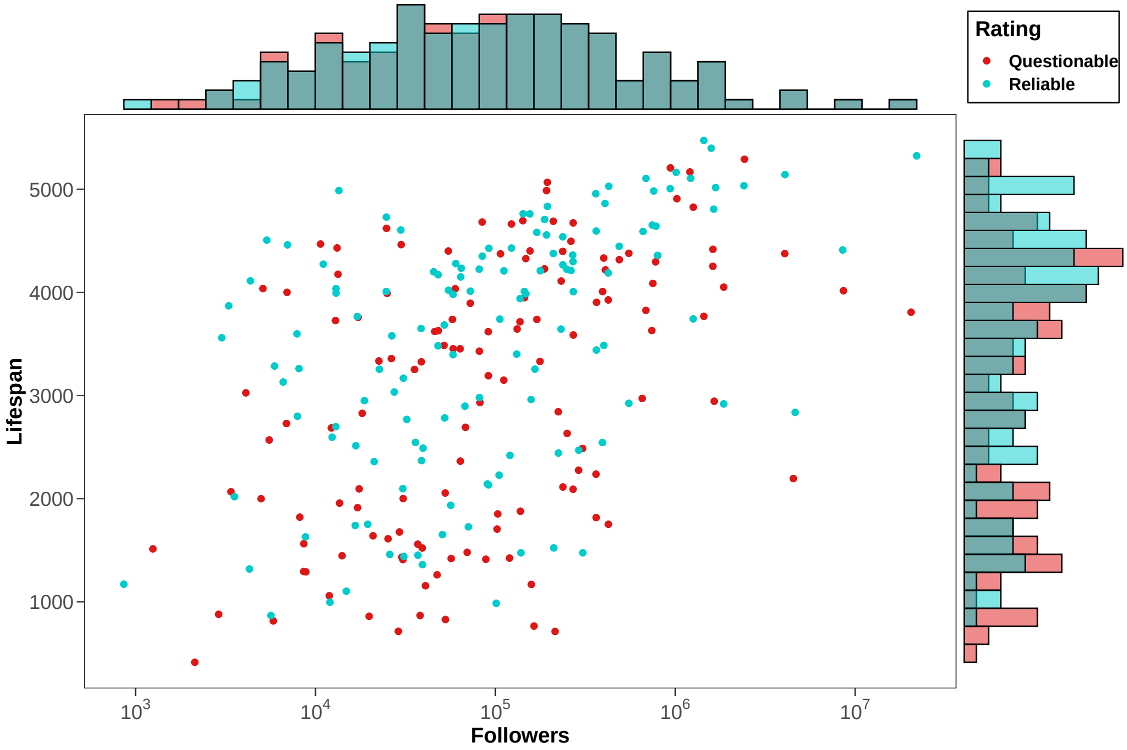

Fig. S9. Scatterplot of Questionable and Reliable pages sub-sample. According to NewsGuard ratings (1), our dataset comprises 898 reliable sources and 131 non-reliable ones. We performed a sampling of 131 reliable sources to have two comparable samples. The resulting sample is obtained by selecting the reliable pages for which the overall Euclidean distance from the non-reliable sample is minimized. We aim to achieve similar structural characteristics, namely Followers and Lifespan (here shown in days). Therefore, we compute distance using the maximum observed number of Followers and the page's creation date as distance variables since most pages' last observations coincide with the end of the analyzed period.

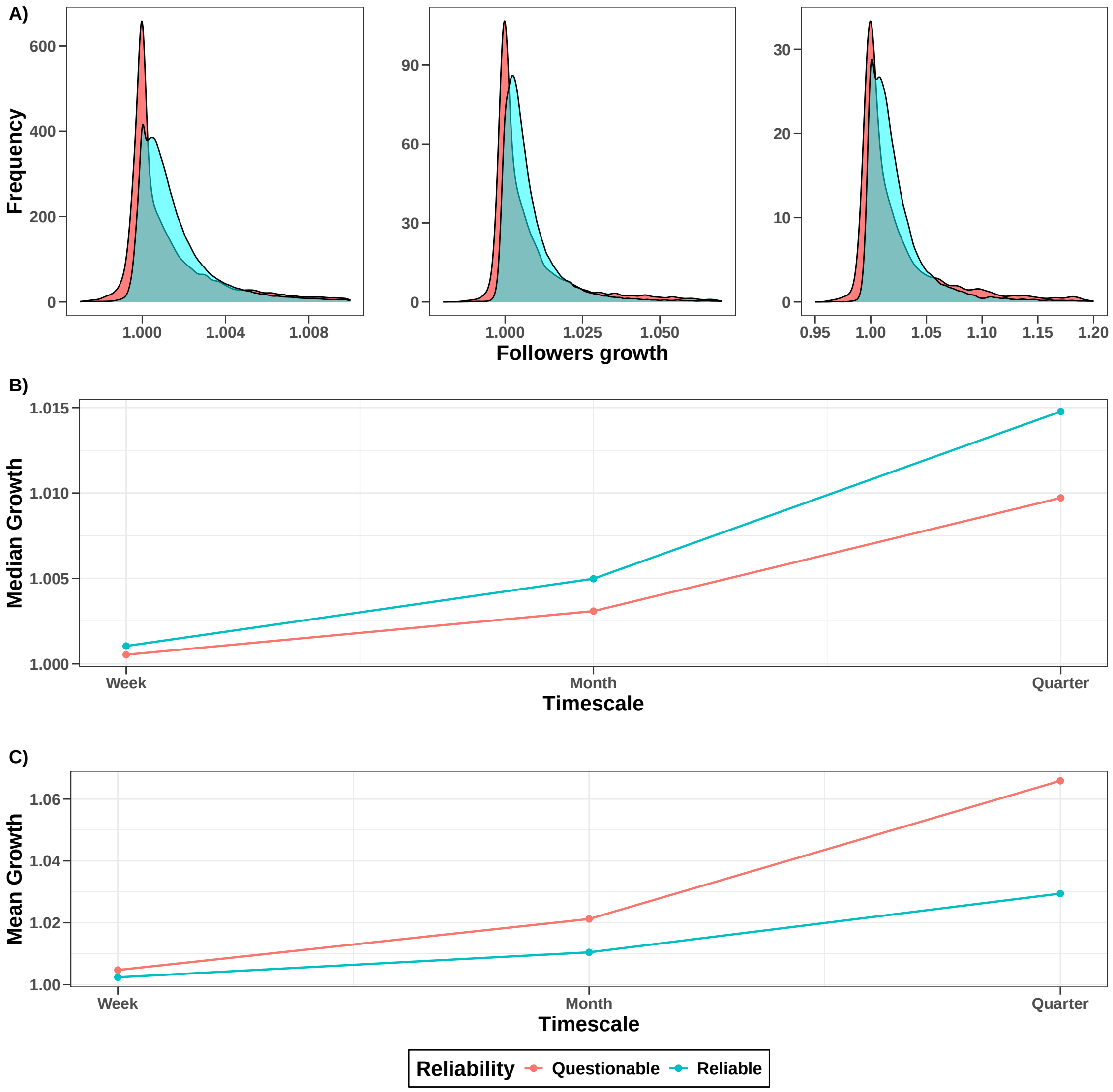

Fig. S10. A) Comparison of Followers growth rate distributions of Questionable and Reliable pages for different timescales. B-C) Median and mean growths of Questionable and Reliable pages across increasing timescales.

Table S1 reports the coefficients of the regression

$$\epsilon_t^{(E)} = \beta_0 + \beta_1 ln(F_t) \quad [1]$$

where $\epsilon_t^{(E)}$ is the logarithm of the Engagement growth rate and $ln(F_t)$ is the logarithm of the Followers value at time t, along with the Pearson Correlation Coefficient for the respective timescale. As results show, both the correlation and the β_1, albeit low, increase by several orders of magnitude with the increase of the timescale. Additionally, we note that in the Daily and Weekly timescales, the p-values of regression coefficients are not significant, as often happens when the coefficients are approximately zero.

	Estimate	Std. Error	t-value	p-value	Pearson Correlation	Timescale
β_0	0.00196	0.00591	0.33118	0.74051	-0.00032	Day
β_1	-0.00020	0.00049	-0.41049	0.68145	-0.00032	Day
β_0	0.01479	0.00926	1.59752	0.11015	-0.00346	Week
β_1	-0.00135	0.00078	-1.73279	0.08313	-0.00346	Week
β_0	0.05231	0.01768	2.95880	0.00309	-0.01328	Month
β_1	-0.00477	0.00149	-3.19999	0.00138	-0.01328	Month
β_0	0.15299	0.03495	4.37747	0.00001	-0.03252	Quarter
β_1	-0.01278	0.00295	-4.33251	0.00001	-0.03252	Quarter

Table S1. Regression coefficients and Pearson Correlation Coefficient between Engagement growth rate and Followers value on increasing timescales.

Table S2 shows p-values of Mann-Whitney U tests (2) between Questionable pages and the sampled subset of Reliable ones. The alternative hypothesis is that the distribution of Reliable sources is right-shifted (higher mean) with respect to the Questionable one. Engagement indicate the absolute value distributions, Engagement growth indicate the growth rate ones. This comparison based on information quality is consistent with overall evidence. The engagement growth of unreliable pages progressively became negative as the time scale widens, in Monthly and Quarterly scales, despite having constantly lower absolute Engagement. Anew, the short-term fluctuations follow a universal dynamic, and neither the reliability turns out to determine growth differences.

Table S3 reports the p-values of the same tests, using the entire set of Reliable pages instead of the sub-sample, showing consistent results.

Table S4 reports the p-values of the tests on Followers' growth, between Questionable pages and the subset of Reliable ones. The alternative hypothesis is that the distribution of Reliable sources is right-shifted (higher mean) with respect to the Questionable one. Engagement indicate the absolute value distributions, Engagement growth indicate the growth rate ones.

Timescale	p-value	Metric
Day	<0.0001	Engagement
Week	<0.0001	Engagement
Month	<0.0001	Engagement
Quarter	<0.0001	Engagement
Day	0.3196	Engagement growth
Week	0.0950	Engagement growth
Month	0.0035	Engagement growth
Quarter	0.0014	Engagement growth

Table S2. p-values of Mann-Whitney U tests between Questionable and the selected subset of Reliable pages. H1: The distribution of Reliable sources is right-shifted (higher mean) with respect to the Questionable one.

Timescale	p-value	Metric
Day	<0.0001	Engagement
Week	<0.0001	Engagement
Month	<0.0001	Engagement
Quarter	<0.0001	Engagement
Day	0.2719	Engagement growth
Week	0.0162	Engagement growth
Month	0.0001	Engagement growth
Quarter	<0.0001	Engagement growth

Table S3. p-values of Mann-Whitney U tests between Questionable and Reliable pages using the entire set of Reliable pages. H1: The distribution of Reliable sources is right-shifted (higher mean) with respect to the Questionable one.

Timescale	p-value	Metric
Week	<0.0001	Followers
Month	<0.0001	Followers
Quarter	<0.0001	Followers
Week	<0.0001	Followers growth
Month	<0.0001	Followers growth
Quarter	<0.0001	Followers growth

Table S4. p-values of Mann-Whitney U tests between Followers growth of Questionable and Reliable pages. H1: The distribution of Reliable sources is right-shifted (higher mean) with respect to the Questionable one.

References

1. Newsguard, 2023. https://www.newsguardtech.com.
2. Henry B Mann and Donald R Whitney. On a test of whether one of two random variables is stochastically larger than the other. *The annals of mathematical statistics*, pages 50–60, 1947.

4. List of Pages

Here we provide the list of pages we use in our analysis. The table reports the page name, its Facebook URL, the language of its contents, and the reliability label for the pages we select in the analysis of Section Growth and Information Quality, by which the latter can be replicated. For replicating all other Sections, it is sufficient to have the data of the pages downloadable through CrowdTangle by using their URLs and following our procedure explained in Section Data Collection in the SI Appendix.

Table S5. List of pages we use in our analysis.

	Page	Facebook	Language	Reliability
1	Armagh I	https://www.facebook.com/armaghonline	eng	
2	Attitude Magazine	https://www.facebook.com/attitude.co.uk	eng	
3	BBC News	https://www.facebook.com/bbcnews	eng	
4	BBC Science Focus Magazine	https://www.facebook.com/sciencefocus	eng	
5	BMJ	https://www.facebook.com/bmjdotcom	eng	
6	Belfast Live	https://www.facebook.com/BelfastLiveOnline	eng	
7	Belfast Telegraph	https://www.facebook.com/belfasttelegraph	eng	
8	Bellingcat	https://www.facebook.com/bellingcat	eng	
9	Berkshire Live	https://www.facebook.com/BerkshireLive	eng	
10	Black Ballad	https://www.facebook.com/blackballadUK	eng	Reliable
11	Bolton News	https://www.facebook.com/theboltonnews	eng	Reliable
12	Bournemouth Daily Echo	https://www.facebook.com/bournemouthdailyecho	eng	
13	BrightVibes	https://www.facebook.com/brightvibes	eng	
14	Bristol.Live	https://www.facebook.com/bristol.live	eng	
15	British GQ	https://www.facebook.com/BritishGQ	eng	
16	Byline Times	https://www.facebook.com/BylineTimes	eng	Reliable
17	Cambridgeshire Live	https://www.facebook.com/cambridgeshirelive	eng	
18	Cancer Research UK	https://www.facebook.com/cancerresearchuk	eng	
19	Channel 4 News	https://www.facebook.com/Channel4News	eng	
20	Cheshire Live	https://www.facebook.com/CheshireLive	eng	
21	City AM	https://www.facebook.com/cityam	eng	
22	Climate Home News	https://www.facebook.com/ClimateHomeNews	eng	
23	Colchester Gazette	https://www.facebook.com/colchester.gazette	eng	Reliable
24	Cork Beo	https://www.facebook.com/corkbeo	eng	
25	CornishStuff.com	https://www.facebook.com/cornishstuff	eng	
26	Cornwall Live	https://www.facebook.com/cornwalllivenews	eng	
27	County Gazette	https://www.facebook.com/CountyGazetteSomerset	eng	
28	Coventry Live	https://www.facebook.com/livecoventry	eng	
29	Daily Echo	https://www.facebook.com/dailyecho	eng	
30	Daily Express	https://www.facebook.com/DailyExpress	eng	

	Page	Facebook	Language	Reliability
31	Daily Mail	https://www.facebook.com/DailyMail	eng	Reliable
32	Daily Record	https://www.facebook.com/TheScottishDailyRecord	eng	
33	Daily Star	https://www.facebook.com/thedailystar	eng	
34	Dazed and Confused Magazine	https://www.facebook.com/DazedandConfusedMagazine	eng	
35	Deeside.com	https://www.facebook.com/Deesidenews	eng	Reliable
36	Derbyshire Live	https://www.facebook.com/derbyshirelive	eng	
37	Derbyshire Times	https://www.facebook.com/derbyshiretimes	eng	
38	Doncaster Free Press	https://www.facebook.com/DonnyFP	eng	
39	Dorset Echo	https://www.facebook.com/dorsetecho	eng	
40	Dublin Live	https://www.facebook.com/DublinLiveOnline	eng	Reliable
41	EDP 24	https://www.facebook.com/edp24	eng	
42	EU Reporter	https://www.facebook.com/eureporter	eng	Questionable
43	East Anglian Daily Times	https://www.facebook.com/eadt24	eng	
44	Echo Essex	https://www.facebook.com/echo.essex	eng	
45	Edinburgh Live	https://www.facebook.com/edinburghlivenews	eng	
46	Essex Live	https://www.facebook.com/essexlivenews	eng	
47	Euronews English	https://www.facebook.com/euronews	eng	Questionable
48	Euronews Italiano	https://www.facebook.com/it.euronews	eng	Questionable
49	European Centre for Disease Prevention and Control	https://www.facebook.com/ECDC.EU	eng	
50	EvolvePolitics	https://www.facebook.com/EvolvePolitics	eng	
51	Express & Star	https://www.facebook.com/ExpressandStar	eng	
52	Families UK	https://www.facebook.com/FamiliesUK	eng	
53	Fife Free Press	https://www.facebook.com/FifeFreePress	eng	
54	Full Fact	https://www.facebook.com/FullFact.org	eng	
55	GAY TIMES	https://www.facebook.com/gaytimes	eng	
56	GB News	https://www.facebook.com/GBNewsOnline	eng	
57	GPonline.com	https://www.facebook.com/gpwebsite	eng	
58	Gedling Eye	https://www.facebook.com/GedlingEye	eng	Reliable
59	Glasgow Live	https://www.facebook.com/glasgowlive	eng	Reliable
60	Glasgow Times	https://www.facebook.com/glasgowtimes	eng	
61	Gloucestershire Live	https://www.facebook.com/GlosLiveOnline	eng	
62	Grimsby Live	https://www.facebook.com/grimsbylive	eng	
63	Guido Fawkes	https://www.facebook.com/fawkespage	eng	Reliable
64	Hampshire Live	https://www.facebook.com/HampshireLiveNews	eng	
65	Health Service Journal	https://www.facebook.com/HSJ.community	eng	Reliable
66	HeraldScotland	https://www.facebook.com/heraldscotland	eng	
67	HuffPost UK	https://www.facebook.com/HuffPostUK	eng	
68	Hull Live	https://www.facebook.com/hulllive	eng	
69	ITV	https://www.facebook.com/itv	eng	Reliable
70	IlmFeed	https://www.facebook.com/ilmfeed	eng	
71	Intelligent Living	https://www.facebook.com/IntelligentLiving	eng	
72	Ipswich Star	https://www.facebook.com/ipswichstar24	eng	
73	Irish News	https://www.facebook.com/IrishNewsLtd	eng	
74	Island Echo	https://www.facebook.com/IslandEcho	eng	
75	Isle of Wight County Press	https://www.facebook.com/iwcponline	eng	Reliable
76	Isle of Wight News from On The Wight	https://www.facebook.com/isleofwightnews	eng	
77	JOE.co.uk	https://www.facebook.com/www.JOE.co.uk	eng	Reliable
78	KentLive	https://www.facebook.com/kentlivenews	eng	
79	KentOnline News	https://www.facebook.com/KentOnline	eng	
80	LADbible	https://www.facebook.com/LADbible	eng	
81	LBC	https://www.facebook.com/LBC	eng	Reliable
82	LabourList	https://www.facebook.com/LabourList	eng	
83	Lancashire Post	https://www.facebook.com/lancashireeveningpost	eng	Reliable
84	Lancashire Telegraph	https://www.facebook.com/lancashiretelegraph	eng	
85	Lancs Live	https://www.facebook.com/lancslive	eng	Reliable
86	Leader Live	https://www.facebook.com/LeaderLive	eng	
87	LeedsLive	https://www.facebook.com/leedslivenews	eng	
88	Leicestershire Live	https://www.facebook.com/leicestershirelive	eng	
89	Lincolnshire Live	https://www.facebook.com/LincsLive	eng	Reliable
90	Liverpool Echo News	https://www.facebook.com/theliverpoolecho	eng	Reliable
91	London Evening Standard	https://www.facebook.com/eveningstandard	eng	
92	London Real	https://www.facebook.com/LondonReal	eng	Questionable
93	Loveworld UK	https://www.facebook.com/loveworldUK	eng	Questionable
94	MIPT	https://www.facebook.com/madeinpoortaste	eng	Questionable
95	Macmillan Cancer Support	https://www.facebook.com/macmillancancer	eng	
96	Manchester Evening News	https://www.facebook.com/ManchesterEveningNews	eng	
97	Mansfield Chad	https://www.facebook.com/mansfieldchad	eng	

	Page	Facebook	Language	Reliability
98	Marie Claire UK	https://www.facebook.com/MarieClaireUK	eng	Reliable
99	Metro	https://www.facebook.com/MetroUK	eng	
100	Middle East Monitor	https://www.facebook.com/middleeastmonitor	eng	Questionable
101	MyLondon	https://www.facebook.com/mylondonnews	eng	Reliable
102	Nation.Cymru	https://www.facebook.com/nation.cymru	eng	Reliable
103	NationalWorld	https://www.facebook.com/NationalWorldUK	eng	
104	Nature	https://www.facebook.com/Nature	eng	
105	New Scientist	https://www.facebook.com/newscientist	eng	
106	Newark Advertiser	https://www.facebook.com/NewarkAdvertiser	eng	
107	Newcastle Chronicle	https://www.facebook.com/NewcastleChronicle	eng	
108	News & Star The Cumberland News	https://www.facebook.com/newsandstar	eng	
109	News Letter	https://www.facebook.com/belfastnewsletter	eng	
110	News Medical	https://www.facebook.com/NewsMedical	eng	Reliable
111	News Shopper	https://www.facebook.com/NewsShopper	eng	Reliable
112	NewsThump	https://www.facebook.com/NewsThump	eng	
113	Newsroom24	https://www.facebook.com/thenewsroom24	eng	Questionable
114	North Wales Live	https://www.facebook.com/northwaleslive	eng	
115	Northampton Chronicle	https://www.facebook.com/northamptonchron	eng	
116	Northants Live	https://www.facebook.com/NorthantsLive	eng	
117	Norwich Evening News	https://www.facebook.com/NorwichEveningNews	eng	
118	Nottinghamshire Live	https://www.facebook.com/nottinghamshirelive	eng	Reliable
119	Nursing Times	https://www.facebook.com/NursingTimesJournal	eng	
120	NursingNotes	https://www.facebook.com/nursingnotesuk	eng	
121	OffGuardian	https://www.facebook.com/offguardian	eng	Questionable
122	Our World in Data	https://www.facebook.com/OurWorldinData	eng	
123	Oxford Mail	https://www.facebook.com/oxfordmail	eng	
124	PETA UK	https://www.facebook.com/OfficialPETAUK	eng	Reliable
125	POLITICO	https://www.facebook.com/politico	eng	
126	Patient	https://www.facebook.com/patient	eng	
127	Peterborough Telegraph	https://www.facebook.com/peterboroughtoday	eng	
128	PinkNews	https://www.facebook.com/pinknews	eng	
129	Plant Based News	https://www.facebook.com/plantbasednews	eng	
130	Plymouth Live	https://www.facebook.com/plymouthlive	eng	
131	Police Hour	https://www.facebook.com/policehour	eng	
132	Political UK News	https://www.facebook.com/politicalukblog	eng	
133	Politicalite	https://www.facebook.com/politicalite	eng	Questionable
134	Politics.co.uk	https://www.facebook.com/politicscoukofficial	eng	
135	PoliticsHome	https://www.facebook.com/PoliticsHomeUK	eng	Reliable
136	Press Gazette	https://www.facebook.com/pressgazette	eng	Reliable
137	Proper Manchester	https://www.facebook.com/ProperMCR	eng	
138	Pulse	https://www.facebook.com/PulseToday.co.uk	eng	Reliable
139	RTÉ	https://www.facebook.com/ExploreRTE	eng	
140	Royal Central	https://www.facebook.com/royalcentral	eng	
141	STV	https://www.facebook.com/WeAreSTV	eng	
142	Scottish Daily Express	https://www.facebook.com/ScottishDailyExpress	eng	
143	Shetland News	https://www.facebook.com/shetlandnews	eng	
144	Shropshire Star	https://www.facebook.com/ShropshireStar	eng	
145	Sick Chirpse	https://www.facebook.com/sickchirpse	eng	Questionable
146	Sky News	https://www.facebook.com/skynews	eng	
147	Somerset Live	https://www.facebook.com/somersetlive	eng	
148	South Wales Argus	https://www.facebook.com/southwalesargus	eng	
149	Staffordshire Live	https://www.facebook.com/staffordshirelive	eng	Reliable
150	Stoke-on-Trent Live	https://www.facebook.com/stokeontrentlive	eng	
151	Stroud News & Journal	https://www.facebook.com/stroudnews	eng	
152	Suffolk Gazette	https://www.facebook.com/suffolkgazette	eng	
153	Suffolk News	https://www.facebook.com/SuffolkNews	eng	Reliable
154	Sunderland Echo	https://www.facebook.com/sunderlandechoonline	eng	
155	Sussex Live	https://www.facebook.com/sussexlive	eng	
156	Swindon Advertiser	https://www.facebook.com/swindonadvertiser	eng	
157	Tatler	https://www.facebook.com/Tatlermagazine	eng	
158	TechRadar	https://www.facebook.com/TechRadar	eng	
159	Technology Networks	https://www.facebook.com/technologynetworks	eng	
160	Tes	https://www.facebook.com/TesNews	eng	
161	The Argus	https://www.facebook.com/brightonargus	eng	
162	The Canary	https://www.facebook.com/TheCanaryUK	eng	
163	The Catholic Herald	https://www.facebook.com/CatholicHeraldMagazine	eng	
164	The Courier UK	https://www.facebook.com/thecourieruk	eng	

	Page	Facebook	Language	Reliability
165	The Daily Mash	https://www.facebook.com/thedailymash	eng	
166	The Drum	https://www.facebook.com/thedrumpage	eng	
167	The Guardian	https://www.facebook.com/theguardian	eng	
168	The Independent	https://www.facebook.com/TheIndependentOnline	eng	
169	The Inverness Courier	https://www.facebook.com/invernesscourier	eng	
170	The Irish Post	https://www.facebook.com/TheIrishPost	eng	
171	The Jewish Chronicle	https://www.facebook.com/jewishchronicle	eng	
172	The Lancet	https://www.facebook.com/TheLancetMedicalJournal	eng	
173	The Lincolnite	https://www.facebook.com/thelincolnite	eng	
174	The London Economic	https://www.facebook.com/TheLondonEconomic	eng	
175	The Mail	https://www.facebook.com/northwesteveningmail	eng	
176	The Manc	https://www.facebook.com/themancuk	eng	
177	The Mirror	https://www.facebook.com/dailymirror	eng	
178	The Morning Star	https://www.facebook.com/morningstaronline	eng	
179	The Muslim News	https://www.facebook.com/themuslimnews	eng	Reliable
180	The National Newspaper	https://www.facebook.com/thenationalnewspaperscotland	eng	
181	The New Arab	https://www.facebook.com/AlAraby.en	eng	
182	The New European	https://www.facebook.com/theneweuropean	eng	
183	The New Statesman	https://www.facebook.com/NewStatesman	eng	
184	The News, Portsmouth	https://www.facebook.com/portsmouthnews	eng	
185	The Northern Echo	https://www.facebook.com/thenorthernecho	eng	
186	The Northern Scot	https://www.facebook.com/NorthernScot	eng	
187	The Pharmaceutical Journal	https://www.facebook.com/PharmaceuticalJournal	eng	
188	The Poke	https://www.facebook.com/PokeHQ	eng	
189	The Scotsman	https://www.facebook.com/TheScotsmanNewspaper	eng	
190	The Scottish Sun	https://www.facebook.com/thescottishsun	eng	
191	The Skwawkbox	https://www.facebook.com/SKWAWKBOX	eng	
192	The Star, Sheffield	https://www.facebook.com/sheffieldstar	eng	
193	The Tab	https://www.facebook.com/TheTabOfficial	eng	
194	The Tablet	https://www.facebook.com/TheCatholicTablet	eng	
195	The Telegraph	https://www.facebook.com/TELEGRAPH.CO.UK	eng	
196	The Telegraph & Argus	https://www.facebook.com/telegraphandargus	eng	
197	The Times and The Sunday Times	https://www.facebook.com/timesandsundaytimes	eng	
198	The Week UK	https://www.facebook.com/theweekuk	eng	
199	The i Paper	https://www.facebook.com/theipaper	eng	
200	Truth Theory	https://www.facebook.com/TruthTheory	eng	
201	Tyla	https://www.facebook.com/Tyla	eng	
202	UK Column	https://www.facebook.com/ukcolumn	eng	Questionable
203	UK Defence Journal	https://www.facebook.com/ukdefencejournal	eng	Reliable
204	UNN	https://www.facebook.com/UnityNewsNetwork	eng	Questionable
205	UnHerd	https://www.facebook.com/unherd	eng	
206	Voice Newspaper	https://www.facebook.com/voicenews	eng	
207	Vox Political	https://www.facebook.com/VoxPolitical	eng	
208	WalesOnline	https://www.facebook.com/WalesOnline	eng	
209	Warrington Guardian	https://www.facebook.com/warringtonguardian	eng	Reliable
210	Waterford Whispers News	https://www.facebook.com/WhispersNews	eng	
211	What Doctors Don't Tell You	https://www.facebook.com/WDDTY	eng	Questionable
212	Wigan Today	https://www.facebook.com/wigan.today	eng	
213	Wiltshire Live	https://www.facebook.com/WiltshireLiveNews	eng	
214	Wings Over Scotland	https://www.facebook.com/WingsOverScotland	eng	Reliable
215	Worcester News	https://www.facebook.com/theworcesternews	eng	
216	Yorkshire Evening Post	https://www.facebook.com/YEP.newspaper	eng	
217	Yorkshire Post	https://www.facebook.com/yorkshirepost.newspaper	eng	Reliable
218	gal-dem.com	https://www.facebook.com/galdemzine	eng	Reliable
219	indy100.com	https://www.facebook.com/meetIndy100	eng	
220	nhs.uk	https://www.facebook.com/NHSwebsite	eng	
221	openDemocracy	https://www.facebook.com/openDemocracy	eng	
222	spiked	https://www.facebook.com/spikedonline	eng	
223	vactruth.com	https://www.facebook.com/vaccinetruth	eng	Questionable
224	20 Minutes	https://www.facebook.com/20minutes	fre	
225	30 Millions d'Amis	https://www.facebook.com/30millionsdamis	fre	Questionable
226	Acrimed	https://www.facebook.com/acrimed	fre	
227	Actu17	https://www.facebook.com/Actu17	fre	
228	ActuSoins	https://www.facebook.com/actusoins	fre	
229	Agence France-Presse	https://www.facebook.com/AFPfra	fre	
230	AgoraVox	https://www.facebook.com/agoravox	fre	
231	Aleteia FR	https://www.facebook.com/Aleteiafr	fre	Reliable

	Page	Facebook	Language	Reliability
232	Alternative Santé	https://www.facebook.com/alternativesante	fre	Questionable
233	Alternatives économiques	https://www.facebook.com/AlternativesEconomiques	fre	
234	Antipresse	https://www.facebook.com/antipresse	fre	Questionable
235	Arrêt sur images	https://www.facebook.com/arretsurimages.net	fre	
236	Astuce	https://www.facebook.com/eddenyaup	fre	Questionable
237	Atlantico.fr	https://www.facebook.com/atlantico.france	fre	
238	BFMTV	https://www.facebook.com/BFMTV	fre	
239	Bio à la Une	https://www.facebook.com/bioalaune	fre	
240	Boulevard Voltaire	https://www.facebook.com/bvoltaire.fr	fre	Questionable
241	Boursorama	https://www.facebook.com/profile.php?id=100063898754011	fre	
242	Breizh-Info	https://www.facebook.com/breizhinformation	fre	Questionable
243	Brut	https://www.facebook.com/brutofficiel	fre	
244	CGTN Français	https://www.facebook.com/CGTNFrancais	fre	Questionable
245	CNETFrance.fr	https://www.facebook.com/cnetfrance	fre	
246	CNEWS	https://www.facebook.com/CNEWSofficiel	fre	
247	CNRS	https://www.facebook.com/cnrs.fr	fre	
248	CaféDéclic	https://www.facebook.com/cafedeclic	fre	
249	Capital	https://www.facebook.com/Capital.fr	fre	
250	Causeur.fr	https://www.facebook.com/causeur	fre	
251	Centre Presse	https://www.facebook.com/centrepresse	fre	
252	Centre Presse Aveyron	https://www.facebook.com/CentrePresseAveyron	fre	
253	Challenges	https://www.facebook.com/challenges	fre	
254	Charente Libre	https://www.facebook.com/Charentelibre16	fre	
255	Closer France	https://www.facebook.com/closerfrance	fre	
256	Complots faciles pour briller en société	https://www.facebook.com/ComplotsFaciles	fre	
257	Contre-info.com	https://www.facebook.com/profile.php?id=100063764962225	fre	Questionable
258	Contrepoints	https://www.facebook.com/Contrepoints	fre	
259	Corse-Matin	https://www.facebook.com/Page.CorseMatin	fre	
260	Courrier international	https://www.facebook.com/courrierinternational.com	fre	
261	Courrier picard	https://www.facebook.com/lecourrierpicard	fre	Reliable
262	Curioctopus.fr	https://www.facebook.com/Curioctopus.fr	fre	
263	DELTA FM	https://www.facebook.com/DeltaFMofficiel	fre	
264	Dakaractu.com	https://www.facebook.com/dakaractupointcom	fre	
265	Demotivateur	https://www.facebook.com/demotivateur	fre	
266	Dernières Nouvelles d'Alsace - DNA	https://www.facebook.com/DNAlsace	fre	
267	Doctissimo	https://www.facebook.com/doctissimo	fre	
268	Donbass Insider	https://www.facebook.com/DonbassInsider	fre	Questionable
269	Dreuz.info	https://www.facebook.com/Dreuz.info	fre	Questionable
270	Droite TV	https://www.facebook.com/profile.php?id=100079848952686	fre	Questionable
271	Dégageons Macron Pour une France Républicaine	https://www.facebook.com/bsda659	fre	
272	E-santé	https://www.facebook.com/Esantefr	fre	
273	Epoch Times Paris	https://www.facebook.com/EpochTimesParis	fre	Questionable
274	Europe 1	https://www.facebook.com/Europe1	fre	
275	ExoPortail	https://www.facebook.com/exoportailofficiel	fre	Questionable
276	FRANCE 24	https://www.facebook.com/FRANCE24	fre	
277	Fabiosa France	https://www.facebook.com/fabiosa.france	fre	Questionable
278	Famille Chrétienne	https://www.facebook.com/famille.chretienne	fre	
279	Fdesouche	https://www.facebook.com/fdesouche	fre	
280	Forbes France	https://www.facebook.com/ForbesFR	fre	
281	Fourchette & Bikini	https://www.facebook.com/fourchetteetbikini	fre	
282	France Bleu	https://www.facebook.com/reseau.francebleu	fre	Reliable
283	France Maghreb2	https://www.facebook.com/FRANCEMAGHREB2	fre	
284	France-Antilles Guadeloupe	https://www.facebook.com/FranceAntillesGuadeloupe	fre	
285	France-Antilles Martinique	https://www.facebook.com/profile.php?id=100064380865866	fre	
286	France-Guyane	https://www.facebook.com/franceguyane	fre	
287	FranceLibre24.net	https://www.facebook.com/FLibre24.net	fre	Questionable
288	FranceSoir	https://www.facebook.com/FranceSoir	fre	Questionable
289	Franceinfo	https://www.facebook.com/franceinfo	fre	
290	Futura - Inspirons l'avenir	https://www.facebook.com/futura.sciences.fr	fre	
291	GEO France	https://www.facebook.com/GEOmagFrance	fre	
292	GQ France	https://www.facebook.com/GQFrance	fre	
293	Gazette Live Montpellier	https://www.facebook.com/lagazettedemontpellier	fre	
294	Gazette Live Nîmes	https://www.facebook.com/lagazettedenimes	fre	
295	Gentside	https://www.facebook.com/Gentside	fre	
296	Gentside Savoir	https://www.facebook.com/GentsideSavoir	fre	
297	Haute Autorité de Santé	https://www.facebook.com/Haute.Autorite.de.Sante	fre	
298	Horizon	https://www.facebook.com/horizonlaradio	fre	

	Page	Facebook	Language	Reliability
299	Info Chrétienne	https://www.facebook.com/InfoChretienne	fre	
300	Inserm	https://www.facebook.com/inserm.fr	fre	
301	Institut Pasteur	https://www.facebook.com/InstitutPasteur	fre	
302	Investig'Action	https://www.facebook.com/InvestigAction	fre	Questionable
303	JDD	https://www.facebook.com/leJDD	fre	
304	Jean Marc Morandini	https://www.facebook.com/jmmorandini	fre	Questionable
305	Jeune Afrique	https://www.facebook.com/jeuneafrique1	fre	
306	Journal L'Alsace	https://www.facebook.com/lalsace.fr	fre	Reliable
307	Journal La Marseillaise	https://www.facebook.com/lamarseillaise.fr	fre	
308	Journal des Femmes Santé	https://www.facebook.com/journaldesfemmes.sante	fre	Reliable
309	Komitid	https://www.facebook.com/Komitid.fr	fre	Reliable
310	L'Aisne Nouvelle	https://www.facebook.com/aisnenouvelle	fre	
311	L'Ardennais	https://www.facebook.com/journal.lardennais	fre	Reliable
312	L'EQUIPE	https://www.facebook.com/lequipe.fr	fre	
313	L'Est Républicain	https://www.facebook.com/estrepublicain	fre	
314	L'Express	https://www.facebook.com/LExpress	fre	
315	L'Important	https://www.facebook.com/limportant.fr	fre	Questionable
316	L'Indépendant	https://www.facebook.com/lindependant.fr	fre	
317	L'Internaute	https://www.facebook.com/linternaute	fre	
318	L'Obs	https://www.facebook.com/lenouvelobservateur	fre	
319	L'Usine Digitale	https://www.facebook.com/LUsineDigitale	fre	
320	L'insoumission	https://www.facebook.com/L.insoumission	fre	Reliable
321	L'union	https://www.facebook.com/journal.lunionlardennais	fre	
322	L'Écho Républicain	https://www.facebook.com/lechorepublicain.fr	fre	
323	La Commère 43	https://www.facebook.com/lacommere43	fre	Reliable
324	La Croix	https://www.facebook.com/lacroix.journal	fre	
325	La DH	https://www.facebook.com/dhnet	fre	
326	La Dépêche du Midi	https://www.facebook.com/ladepechedumidi	fre	
327	La Lettre Patriote	https://www.facebook.com/lalettrepatriote	fre	Questionable
328	La Libre	https://www.facebook.com/lalibre.be	fre	
329	La Manche Libre	https://www.facebook.com/lamanchelibre	fre	
330	La Montagne	https://www.facebook.com/journal.LaMontagne	fre	
331	La Nouvelle République	https://www.facebook.com/lanouvellerepublique	fre	
332	La Nouvelle République des Pyrénées	https://www.facebook.com/nrpyrenees	fre	
333	La Relève et La Peste	https://www.facebook.com/lareleveetlapeste	fre	
334	La République des Pyrénées	https://www.facebook.com/LaRepubliqueDesPyrenees	fre	
335	La République du Centre	https://www.facebook.com/republiqueducentre	fre	
336	La Semaine dans le Boulonnais	https://www.facebook.com/lasemainedansleboulonnais	fre	
337	La Tribune	https://www.facebook.com/latribune	fre	
338	La gauche m'a tuer	https://www.facebook.com/lagauchematuer1	fre	Questionable
339	LaNutrition.fr	https://www.facebook.com/lanutrition	fre	
340	Lanceur d'alerte.	https://www.facebook.com/LanceurdAlerteOfficial	fre	Questionable
341	Le Berry Républicain	https://www.facebook.com/BerryRepublicain	fre	
342	Le Bien Public	https://www.facebook.com/BienPublic21	fre	
343	Le Courrier des Stratèges	https://www.facebook.com/CourrierDS	fre	Questionable
344	Le Dauphiné Libéré	https://www.facebook.com/ledauphinelibere	fre	
345	Le Figaro	https://www.facebook.com/lefigaro	fre	
346	Le JSL	https://www.facebook.com/LeJSL71	fre	
347	Le Journal du Centre	https://www.facebook.com/journalducentre	fre	
348	Le Messager Chablais	https://www.facebook.com/LeMessagerChablais	fre	
349	Le Monde	https://www.facebook.com/lemonde.fr	fre	
350	Le Monde Juif .info	https://www.facebook.com/LeMondeJuif.info	fre	Questionable
351	Le Média en 4-4-2	https://www.facebook.com/LeMediaEn442	fre	
352	Le Média pour Tous - Vincent Lapierre	https://www.facebook.com/LeMediaPourTous	fre	Questionable
353	Le Nouvel Ordre Mondial	https://www.facebook.com/profile.php?id=100063575413337	fre	
354	Le Parisien	https://www.facebook.com/leparisien	fre	Reliable
355	Le Petit Bleu d'Agen	https://www.facebook.com/lepetitbleudagen	fre	Reliable
356	Le Point	https://www.facebook.com/lepoint.fr	fre	
357	Le Populaire du Centre	https://www.facebook.com/populaireducentre	fre	
358	Le Progrès	https://www.facebook.com/leprogres	fre	
359	Le Quotidien du Médecin	https://www.facebook.com/lequotidiendumedecin.fr	fre	Reliable
360	Le Quotidien du Pharmacien	https://www.facebook.com/lequotidiendupharmacien	fre	
361	Le Républicain Lorrain	https://www.facebook.com/republicainlorrain	fre	Reliable
362	Le Soir	https://www.facebook.com/lesoirbe	fre	Reliable
363	Le Télégramme	https://www.facebook.com/letelegramme	fre	
364	Le Vif	https://www.facebook.com/Leviflexpress	fre	
365	Lelibrepenseur.org	https://www.facebook.com/LeLibrePenseur.org	fre	Questionable

	Page	Facebook	Language	Reliability
366	Les Crieurs du Web	https://www.facebook.com/lescrieursduweb	fre	Questionable
367	Les Echos	https://www.facebook.com/lesechos	fre	
368	Les Numériques	https://www.facebook.com/LesNumeriques	fre	
369	Les-Crises	https://www.facebook.com/les.crises	fre	
370	Libération	https://www.facebook.com/Liberation	fre	Reliable
371	Lyon Capitale	https://www.facebook.com/LyonCap	fre	
372	Lyonmag	https://www.facebook.com/lyonmag	fre	
373	M Radio	https://www.facebook.com/mradiofr	fre	
374	Made in Marseille	https://www.facebook.com/mademarseillenet	fre	Reliable
375	Magazine NEXUS	https://www.facebook.com/magazine.nexus	fre	Questionable
376	Marianne	https://www.facebook.com/Marianne.magazine	fre	
377	Marie Claire France	https://www.facebook.com/marieclairefrance	fre	
378	Marsactu	https://www.facebook.com/marsactu	fre	
379	Maville.com	https://www.facebook.com/Mavillecom	fre	
380	Mediapart	https://www.facebook.com/Mediapart.fr	fre	Reliable
381	Medisite.fr	https://www.facebook.com/medisite.fr	fre	Reliable
382	Midi Libre	https://www.facebook.com/midilibre	fre	
383	Minurne Résistance - Covid19 Justice	https://www.facebook.com/Minurne	fre	Questionable
384	Mona FM	https://www.facebook.com/monafmnord	fre	
385	Mr Mondialisation	https://www.facebook.com/M.Mondialisation	fre	Questionable
386	NOFI	https://www.facebook.com/bignofi	fre	
387	Nice-Matin	https://www.facebook.com/Page.NiceMatin	fre	
388	Nord éclair	https://www.facebook.com/nordeclair.info	fre	
389	Numerama	https://www.facebook.com/Numerama	fre	
390	OISE HEBDO	https://www.facebook.com/oisehebdo	fre	
391	OMG.	https://www.facebook.com/LeMagOMG	fre	Questionable
392	ObjectifGard	https://www.facebook.com/objectifgard	fre	
393	Oh My Mag	https://www.facebook.com/Ohmymag	fre	
394	One Voice	https://www.facebook.com/onevoiceanimal	fre	
395	Opinion Internationale	https://www.facebook.com/OpinionInternationale	fre	Reliable
396	Ouest France	https://www.facebook.com/ouestfrance	fre	
397	Oumma.com	https://www.facebook.com/oummacom	fre	Questionable
398	Page du Yéti	https://www.facebook.com/yetiblog	fre	Questionable
399	Panamza	https://www.facebook.com/panhamza	fre	Questionable
400	Paris Normandie	https://www.facebook.com/parisnormandie	fre	
401	Politis.fr	https://www.facebook.com/Politismag	fre	
402	Pourquoi docteur	https://www.facebook.com/Pourquoidoc	fre	
403	Press TV	https://www.facebook.com/PRESSTV	fre	Questionable
404	Protège ta santé naturellement	https://www.facebook.com/protegetasante	fre	Questionable
405	Prévention Santé	https://www.facebook.com/preventionsante	fre	
406	Psychologies	https://www.facebook.com/Psychologies	fre	
407	Public	https://www.facebook.com/publicfr	fre	
408	Public Sénat	https://www.facebook.com/publicsenat	fre	
409	Puremédias	https://www.facebook.com/puremedias	fre	Reliable
410	Quotidien du Peuple	https://www.facebook.com/profile.php?id=100068655228349	fre	Questionable
411	RT France	https://www.facebook.com/RTFrance	fre	Questionable
412	RTBF Info	https://www.facebook.com/rtbfinfo	fre	
413	RTL	https://www.facebook.com/RTL	fre	
414	RTL info.	https://www.facebook.com/RTLInfo	fre	
415	Radio 6	https://www.facebook.com/radio6officiel	fre	
416	Radio Classique	https://www.facebook.com/R.Classique	fre	
417	Radio Contact • FR	https://www.facebook.com/radiocontactfm	fre	
418	Radio Courtoisie	https://www.facebook.com/radiocourtoisie	fre	Questionable
419	Radio France	https://www.facebook.com/radiofrance	fre	
420	Radio Free Dom	https://www.facebook.com/974radiofreedom	fre	
421	Radio Okapi	https://www.facebook.com/radiookapi	fre	
422	Radio1 Tahiti	https://www.facebook.com/radio1tahiti	fre	
423	RegardeCetteVideo	https://www.facebook.com/regardecettevideo	fre	
424	Reporterre, le média de l'écologie	https://www.facebook.com/ReporterreQuotidienDeLecologie	fre	
425	Riposte Laïque	https://www.facebook.com/RiposteLaique	fre	Questionable
426	Révolution Permanente	https://www.facebook.com/RevolutionPermanente.fr	fre	
427	Sain et Naturel	https://www.facebook.com/SainetNaturel	fre	
428	Santé et beauté pour tous!!	https://www.facebook.com/sante.beaute.tous	fre	Questionable
429	Santé publique France	https://www.facebook.com/santepubliquefrance	fre	Reliable
430	Santé+ Magazine	https://www.facebook.com/santeplusmag	fre	Questionable
431	Savoir Aimer,	https://www.facebook.com/SavoirAimer.off	fre	Questionable
432	Science & Vie	https://www.facebook.com/ScienceetVieMag	fre	

	Page	Facebook	Language	Reliability
433	ScienceInfo	https://www.facebook.com/ScienceInfoOfficiel	fre	
434	Sciencepost	https://www.facebook.com/sciencepost.fr	fre	
435	Sciences et Avenir	https://www.facebook.com/sciencesetavenir	fre	
436	Siècle Digital	https://www.facebook.com/siecledigital	fre	
437	Soignants pour une politique sanitaire juste et proportionnée	https://www.facebook.com/CollectifReinfoCovid	fre	Questionable
438	Sputnik France	https://www.facebook.com/sputnik.france	fre	Questionable
439	Stratégies	https://www.facebook.com/strategies.fr	fre	
440	Sud Radio	https://www.facebook.com/SudRadioOfficiel	fre	Questionable
441	SudOuest.fr	https://www.facebook.com/journalsudouest	fre	
442	TRT Français	https://www.facebook.com/TRT.Francais	fre	
443	TV5MONDE	https://www.facebook.com/tv5mondeofficiel	fre	
444	Tahiti Infos	https://www.facebook.com/Tahitiinfos	fre	Reliable
445	Tendance Ouest	https://www.facebook.com/tendanceouest	fre	
446	Terrafemina	https://www.facebook.com/terrafemina	fre	
447	Ton Mag France	https://www.facebook.com/tonmag3	fre	Questionable
448	Top Santé	https://www.facebook.com/topsantecom	fre	Questionable
449	TousPerfect	https://www.facebook.com/tousperfect	fre	Questionable
450	Trust My Science par J. Paiano	https://www.facebook.com/TrustMyScience	fre	
451	TÊTU	https://www.facebook.com/tetumag	fre	
452	Télérama	https://www.facebook.com/Telerama	fre	
453	Ulyces	https://www.facebook.com/ulyces.co	fre	
454	VICE France	https://www.facebook.com/VICEFrance	fre	
455	VIDAL	https://www.facebook.com/VIDALfrance	fre	
456	Vanity Fair France	https://www.facebook.com/vanityfairfr	fre	Reliable
457	Var-Matin	https://www.facebook.com/Page.VarMatin	fre	
458	Voici	https://www.facebook.com/voici	fre	
459	Vosges Matin	https://www.facebook.com/vosgesmatin	fre	
460	ZDNet.fr	https://www.facebook.com/zdnet.fr	fre	
461	actu.fr	https://www.facebook.com/actu.fr	fre	Reliable
462	al Nas	https://www.facebook.com/alnasfr	fre	Questionable
463	faitsdivers.org	https://www.facebook.com/faitsdivers.org	fre	
464	il était une pub	https://www.facebook.com/Iletaitunepub	fre	Questionable
465	l'Humanité	https://www.facebook.com/humanite.fr	fre	
466	l'Opinion	https://www.facebook.com/lopinionfr	fre	
467	l'est-éclair	https://www.facebook.com/lesteclair	fre	
468	lavenir.net	https://www.facebook.com/lavenir.net	fre	Reliable
469	20 Minuten	https://www.facebook.com/20min	ger	
470	ANTENNE BAYERN	https://www.facebook.com/antennebayern	ger	
471	ARD Mediathek & Das Erste	https://www.facebook.com/DasErste	ger	
472	AZ-Online	https://www.facebook.com/az.online	ger	Reliable
473	Aachener Zeitung	https://www.facebook.com/aachenerzeitung	ger	
474	Abendzeitung München	https://www.facebook.com/abendzeitung.muenchen	ger	
475	Achgut.com - Die Achse des Guten	https://www.facebook.com/achgut	ger	Questionable
476	Alexx	https://www.facebook.com/altermedzentrum	ger	Questionable
477	Allgemeine Zeitung	https://www.facebook.com/allgemeinezeitung	ger	
478	Anti-Spiegel	https://www.facebook.com/AntiSpiegel	ger	Questionable
479	Apolut	https://www.facebook.com/apolut	ger	
480	Apotheken Umschau	https://www.facebook.com/Apotheken.Umschau	ger	Reliable
481	Augsburger Allgemeine	https://www.facebook.com/AugsburgerAllgemeine	ger	
482	B.Z.	https://www.facebook.com/B.Z.Berlin	ger	
483	BR - Bayerischer Rundfunk	https://www.facebook.com/bayerischer.rundfunk	ger	
484	BW24	https://www.facebook.com/bw24news	ger	Reliable
485	Badische Zeitung	https://www.facebook.com/badischezeitung.de	ger	
486	Berliner Kurier	https://www.facebook.com/bkurier	ger	
487	Berliner Morgenpost	https://www.facebook.com/morgenpost	ger	
488	Berliner Zeitung	https://www.facebook.com/berlinerzeitung	ger	
489	Braunschweiger Zeitung	https://www.facebook.com/braunschweigerzeitung	ger	
490	Bundesministerium für Gesundheit	https://www.facebook.com/bmg.bund	ger	
491	Business Insider Deutschland	https://www.facebook.com/Business.Insider.Deutschland	ger	
492	BuzzFeed Deutschland	https://www.facebook.com/BuzzFeedDeutschland	ger	
493	CAPITAL	https://www.facebook.com/capital	ger	
494	Cellesche Zeitung	https://www.facebook.com/CellescheZeitung	ger	Reliable
495	Cicero	https://www.facebook.com/CiceroMagazin	ger	
496	Cuxhavener Nachrichten	https://www.facebook.com/CuxhavenerNachrichten	ger	
497	DER AKTIONÄR	https://www.facebook.com/aktionaer	ger	
498	DER SPIEGEL	https://www.facebook.com/derspiegel	ger	
499	DIE RHEINPFALZ	https://www.facebook.com/rheinpfalz	ger	

	Page	Facebook	Language	Reliability
500	DNN Dresdner Neueste Nachrichten	https://www.facebook.com/dnn.de	ger	
501	DW Communications	https://www.facebook.com/dw.deutschewelle	ger	
502	DW Deutsch	https://www.facebook.com/dw.german	ger	
503	DW Deutsch lernen	https://www.facebook.com/dw.learngerman	ger	
504	DW News	https://www.facebook.com/deutschewellenews	ger	
505	Der Postillon	https://www.facebook.com/DerPostillon	ger	
506	Der Westen	https://www.facebook.com/derwesten.de	ger	
507	Der Wächter	https://www.facebook.com/waechter.investigativ	ger	Questionable
508	Deutsche Apotheker Zeitung	https://www.facebook.com/DAZ.online	ger	
509	Deutsche Wirtschafts Nachrichten	https://www.facebook.com/DeutscheWirtschaftsNachrichten	ger	
510	Deutscher Hanfverband (DHV)	https://www.facebook.com/hanfverband	ger	
511	Deutschland Kurier	https://www.facebook.com/DK.Nachrichten	ger	Questionable
512	Deutschlandfunk	https://www.facebook.com/deutschlandfunk	ger	
513	Deutschlandfunk Kultur	https://www.facebook.com/DLFKultur	ger	
514	Dewezet	https://www.facebook.com/Dewezet.de	ger	
515	DieUnbestechlichen.com	https://www.facebook.com/dieUnbestechlichenNews	ger	Questionable
516	Döbelner Allgemeine Zeitung	https://www.facebook.com/DoebelnerAllgemeineZeitung	ger	
517	EXPRESS	https://www.facebook.com/EXPRESS.Koeln	ger	Reliable
518	Echo Online	https://www.facebook.com/echoonline	ger	
519	FAZ.NET - Frankfurter Allgemeine Zeitung	https://www.facebook.com/faz	ger	
520	FIT FOR FUN	https://www.facebook.com/FitForFun.de	ger	
521	FNP - Frankfurter Neue Presse	https://www.facebook.com/FrankfurterNeuePresse	ger	
522	FOCUS online	https://www.facebook.com/focus.de	ger	
523	Faktastisch	https://www.facebook.com/faktastisch	ger	
524	Frankenpost	https://www.facebook.com/Frankenpost	ger	
525	Frankfurter Rundschau	https://www.facebook.com/FrankfurterRundschau	ger	
526	Freie Medien	https://www.facebook.com/FreieMedien2.0	ger	Questionable
527	Freie Presse	https://www.facebook.com/freiepresse	ger	
528	GMX News	https://www.facebook.com/GMX.DE	ger	
529	General-Anzeiger Bonn	https://www.facebook.com/gaonline	ger	
530	Gießener Allgemeine Zeitung	https://www.facebook.com/GiessenerAllgemeine	ger	
531	Golem.de	https://www.facebook.com/golem	ger	
532	Grafschafter Nachrichten	https://www.facebook.com/grafschafter.nachrichten	ger	
533	Göttinger Tageblatt	https://www.facebook.com/goettingertageblatt	ger	
534	HNA	https://www.facebook.com/HNA	ger	
535	Hamburger Abendblatt	https://www.facebook.com/abendblatt	ger	
536	Hamburger Morgenpost	https://www.facebook.com/hamburgermorgenpost	ger	
537	Handelsblatt	https://www.facebook.com/handelsblatt	ger	
538	Hanf Journal	https://www.facebook.com/hanfjournal	ger	
539	Hannoversche Allgemeine Zeitung HAZ	https://www.facebook.com/HannoverscheAllgemeine	ger	
540	Heidelberg24	https://www.facebook.com/heidelberg24	ger	
541	Heilbronner Stimme	https://www.facebook.com/HeilbronnerStimme	ger	
542	Heilpraxisnet.de	https://www.facebook.com/Heilpraxis	ger	
543	JETZT	https://www.facebook.com/jetzt.de	ger	
544	JUNGE FREIHEIT	https://www.facebook.com/jungefreiheit	ger	
545	Jüdische Allgemeine	https://www.facebook.com/JuedischeAllgemeine	ger	
546	KSTA	https://www.facebook.com/ksta	ger	
547	KSTA Rhein-Erft	https://www.facebook.com/ksta.rhein.erft	ger	
548	Kieler Nachrichten	https://www.facebook.com/kielernachrichten	ger	
549	Kopp Verlag	https://www.facebook.com/koppverlag.de	ger	Questionable
550	Kölnische Rundschau	https://www.facebook.com/rundschau.online	ger	
551	LVZ Kreis Leipzig	https://www.facebook.com/lvz.kreisleipzig	ger	
552	LVZ Leipziger Volkszeitung	https://www.facebook.com/lvz.de	ger	
553	LVZ Nordsachsen	https://www.facebook.com/lvz.nordsachsen	ger	
554	LVZ Sport	https://www.facebook.com/lvzsport	ger	
555	Landeszeitung Lüneburg	https://www.facebook.com/landeszeitung	ger	
556	Lausitzer Rundschau	https://www.facebook.com/lausitzerrundschau	ger	
557	Lokalzeit	https://www.facebook.com/wdrlokalzeit	ger	
558	Ludwigsburger Kreiszeitung www.lkz.de	https://www.facebook.com/ludwigsburger.kreiszeitung	ger	
559	Lübecker Nachrichten Online	https://www.facebook.com/LNOnline	ger	
560	Lüdenscheider Nachrichten	https://www.facebook.com/luedenscheider.nachrichten	ger	Reliable
561	MAZonline Märkische Allgemeine	https://www.facebook.com/MAZonline	ger	
562	MDR - Mitteldeutscher Rundfunk	https://www.facebook.com/mdr	ger	
563	Main-Post	https://www.facebook.com/mainpost	ger	
564	Mannheimer Morgen	https://www.facebook.com/mannheimer.morgen	ger	
565	Merkur.de	https://www.facebook.com/merkuronline	ger	
566	Mittelbadische Presse - Zeitungen der Ortenau	https://www.facebook.com/mittelbadische.presse	ger	

	Page	Facebook	Language	Reliability
567	Mittelbayerische	https://www.facebook.com/mittelbayerische	ger	
568	Mitteldeutsche Zeitung	https://www.facebook.com/mzwebde	ger	
569	Märkische Onlinezeitung MOZ.de	https://www.facebook.com/mozde	ger	
570	NDR.de	https://www.facebook.com/NDR.de	ger	
571	NEOPresse - Unabhängige Nachrichten	https://www.facebook.com/neopr	ger	Questionable
572	NRZ	https://www.facebook.com/NRZ.de	ger	
573	NWZonline	https://www.facebook.com/nwzonline	ger	
574	NZZ Neue Zürcher Zeitung	https://www.facebook.com/nzz	ger	
575	NachDenkSeiten	https://www.facebook.com/NachDenkSeiten	ger	
576	NetDoktor.de	https://www.facebook.com/NetDoktor.de	ger	
577	Netzfrauen	https://www.facebook.com/Netzfrauen	ger	
578	Neue Presse Hannover NP	https://www.facebook.com/neuepresse	ger	
579	Neue Westfälische	https://www.facebook.com/NeueWestfaelische	ger	
580	News4teachers	https://www.facebook.com/News4teachers	ger	
581	Niederelbe-Zeitung	https://www.facebook.com/NiederelbeZeitung	ger	
582	Nordbayerischer Kurier	https://www.facebook.com/Nordbayerischer.Kurier	ger	
583	Nordkurier	https://www.facebook.com/Nordkurier	ger	
584	Nürnberger Nachrichten	https://www.facebook.com/Nuernberger.Nachrichten.Online	ger	
585	OTZ	https://www.facebook.com/otz.de	ger	
586	Oberhessische Presse	https://www.facebook.com/OberhessischePresse	ger	
587	Onetz	https://www.facebook.com/onetz.de	ger	
588	Onmeda	https://www.facebook.com/onmeda	ger	
589	Ostsee-Zeitung	https://www.facebook.com/Ostseezeitung	ger	
590	Ostsee-Zeitung Usedom	https://www.facebook.com/oz.usedom	ger	Reliable
591	PZ-news.de	https://www.facebook.com/pznews	ger	
592	Passauer Neue Presse - PNP	https://www.facebook.com/pnp.de	ger	
593	Pharmazeutische Zeitung	https://www.facebook.com/PharmazeutischeZeitung	ger	
594	Potsdamer Neueste Nachrichten	https://www.facebook.com/PNN.de	ger	
595	Praxisvita.de	https://www.facebook.com/praxisvita.de	ger	
596	QS24	https://www.facebook.com/qs24official	ger	Questionable
597	Quarks	https://www.facebook.com/quarks.de	ger	
598	RND - RedaktionsNetzwerk Deutschland	https://www.facebook.com/RedaktionsNetzwerkDeutschland	ger	
599	RPR1.	https://www.facebook.com/radiorpr1	ger	
600	RT DE	https://www.facebook.com/rtde	ger	Questionable
601	Radio Brocken	https://www.facebook.com/RadioBrocken	ger	
602	Radio Regenbogen	https://www.facebook.com/radioregenbogen	ger	
603	Rhein-Neckar-Zeitung	https://www.facebook.com/RheinNeckarZeitung	ger	Reliable
604	Rhein-Zeitung	https://www.facebook.com/rheinzeitung	ger	
605	Rheinische Post	https://www.facebook.com/rponline	ger	
606	Rolling Stone Magazin	https://www.facebook.com/rollingstonemagazin	ger	
607	Ruhr Nachrichten	https://www.facebook.com/RuhrNachrichten	ger	
608	SACHSEN FERNSEHEN Chemnitz	https://www.facebook.com/chemnitzfernsehen	ger	
609	SACHSEN FERNSEHEN Dresden	https://www.facebook.com/dresdenfernsehen	ger	
610	SACHSEN FERNSEHEN Leipzig	https://www.facebook.com/leipzigfernsehen	ger	
611	SACHSEN FERNSEHEN Vogtland	https://www.facebook.com/sachsenfernsehen.vogtland	ger	Reliable
612	SNA	https://www.facebook.com/snanews.de	ger	Questionable
613	SOL.DE - Saarland Online	https://www.facebook.com/saarlandonline	ger	Reliable
614	SR.de	https://www.facebook.com/SRonline.de	ger	
615	SRF Schweizer Radio und Fernsehen	https://www.facebook.com/srf.ch	ger	
616	SWR Aktuell	https://www.facebook.com/SWRAktuell	ger	
617	SWR Wissen	https://www.facebook.com/SWRWissen	ger	Reliable
618	SWR3	https://www.facebook.com/SWR3	ger	
619	Saarbrücker Zeitung	https://www.facebook.com/saarbrueckerzeitung	ger	
620	Schaumburger Nachrichten	https://www.facebook.com/SchaumburgerNachrichten	ger	
621	Schwarzwälder Bote	https://www.facebook.com/SchwarzwaelderBote	ger	
622	Schwäbische	https://www.facebook.com/schwaebische.de	ger	
623	Siegener Zeitung	https://www.facebook.com/siegenerzeitung	ger	
624	Soester Anzeiger	https://www.facebook.com/soester.anzeiger	ger	
625	Spektrum der Wissenschaft	https://www.facebook.com/spektrumverlag	ger	
626	Stuttgarter Nachrichten	https://www.facebook.com/stuttgarternachrichten	ger	
627	Stuttgarter Zeitung	https://www.facebook.com/stuttgarterzeitung	ger	
628	Sächsische.de	https://www.facebook.com/saechsische.de	ger	
629	Süddeutsche Zeitung	https://www.facebook.com/ihre.sz	ger	
630	Südwest Presse	https://www.facebook.com/swp.de	ger	
631	TA Thüringer Allgemeine	https://www.facebook.com/thueringerallgemeine	ger	
632	TAG24	https://www.facebook.com/tag24.deutschland	ger	Reliable
633	TAG24 Bielefeld	https://www.facebook.com/tag24.bielefeld	ger	Reliable

	Page	Facebook	Language	Reliability
634	Tages-Anzeiger	https://www.facebook.com/Tagesanzeiger	ger	
635	Tagesspiegel	https://www.facebook.com/Tagesspiegel	ger	
636	Thüringen24	https://www.facebook.com/thueringen24	ger	Reliable
637	Thüringer Allgemeine Erfurt	https://www.facebook.com/taerfurt	ger	
638	Thüringer Allgemeine Kyffhäuserkreis	https://www.facebook.com/kyffhaeuserkreis	ger	
639	Thüringer Allgemeine Sömmerda	https://www.facebook.com/tasoemm	ger	
640	Thüringer Allgemeine Weimar	https://www.facebook.com/TA.Weimar	ger	Reliable
641	Thüringische Landeszeitung - TLZ	https://www.facebook.com/tlz.de	ger	
642	Tichys Einblick	https://www.facebook.com/tichyseinblick	ger	Reliable
643	Unsere Natur	https://www.facebook.com/unserenatur369	ger	Questionable
644	Utopia	https://www.facebook.com/utopia.de	ger	
645	VICE auf Deutsch	https://www.facebook.com/vicede	ger	
646	VOGUE Germany	https://www.facebook.com/VOGUEDeutschland	ger	
647	Volksstimme.de	https://www.facebook.com/volksstimme	ger	
648	Volksverpetzer	https://www.facebook.com/volksverpetzer	ger	
649	WA	https://www.facebook.com/westfaelischer.anzeiger	ger	
650	WAZ	https://www.facebook.com/WAZRedaktion	ger	
651	WAZ Bochum	https://www.facebook.com/WAZBochum	ger	
652	WAZ Bottrop	https://www.facebook.com/WAZBottrop	ger	
653	WAZ Duisburg	https://www.facebook.com/WAZDuisburg	ger	
654	WAZ Essen	https://www.facebook.com/WAZEssen	ger	
655	WAZ Gelsenkirchen	https://www.facebook.com/WAZGelsenkirchen	ger	
656	WAZ Mülheim	https://www.facebook.com/WAZMuelheim	ger	
657	WAZ Witten	https://www.facebook.com/WAZWitten	ger	
658	WAZ Wolfsburger Allgemeine Zeitung	https://www.facebook.com/wolfsburgerallgemeine	ger	
659	WDR	https://www.facebook.com/WDR	ger	
660	WELT	https://www.facebook.com/welt	ger	
661	WESER-KURIER	https://www.facebook.com/WESER.KURIER	ger	
662	WZ - Westdeutsche Zeitung	https://www.facebook.com/wznewsline	ger	Reliable
663	Westfalen-Blatt	https://www.facebook.com/westfalenblatt	ger	
664	Westfalenpost Altkreis Brilon	https://www.facebook.com/WestfalenpostBrilon	ger	
665	Westfalenpost Hagen	https://www.facebook.com/WestfalenpostHagen	ger	
666	Westfalenpost Kreis Olpe	https://www.facebook.com/WestfalenpostOlpe	ger	
667	Westfalenpost Meschede	https://www.facebook.com/wp.meschede	ger	
668	Westfalenpost Wittgenstein	https://www.facebook.com/WestfalenpostWittgenstein	ger	Reliable
669	Westfälische Nachrichten	https://www.facebook.com/wnonline	ger	
670	Wiesbadener Kurier	https://www.facebook.com/wiesbadenerkurier	ger	
671	WirtschaftsWoche	https://www.facebook.com/Wirtschaftswoche	ger	
672	ZDF	https://www.facebook.com/ZDF	ger	
673	ZDF Magazin Royale	https://www.facebook.com/zdfmagazinroyale	ger	
674	ZDF heute	https://www.facebook.com/ZDFheute	ger	
675	ZDF heute-show	https://www.facebook.com/heuteshow	ger	
676	ZEIT ONLINE	https://www.facebook.com/zeitonline	ger	
677	ZUERST! – Deutsches Nachrichtenmagazin	https://www.facebook.com/ZUERST.Magazin	ger	Questionable
678	Zeitungsverlag Waiblingen	https://www.facebook.com/zvwonline	ger	
679	Zentrum der Gesundheit	https://www.facebook.com/ZentrumDerGesundheit	ger	Questionable
680	aerzteblatt.de	https://www.facebook.com/aerzteblatt	ger	
681	agrarheute	https://www.facebook.com/agrarheutecom	ger	
682	all-in.de - das Allgäu online.	https://www.facebook.com/allin.de.dasallgaeuonline	ger	
683	bild der wissenschaft	https://www.facebook.com/bildderwissenschaft	ger	
684	bnn.de Badische Neueste Nachrichten	https://www.facebook.com/badischeneuestenachrichten	ger	
685	buten un binnen	https://www.facebook.com/butenunbinnen	ger	Reliable
686	der Freitag	https://www.facebook.com/derfreitag	ger	
687	donaukurier	https://www.facebook.com/donaukurier.online	ger	
688	eigentümlich frei	https://www.facebook.com/efmagazin	ger	Questionable
689	fudder.de	https://www.facebook.com/fudder.de	ger	
690	heise online	https://www.facebook.com/heiseonline	ger	
691	hessenschau	https://www.facebook.com/Hessenschau	ger	
692	hildesheimer-allgemeine.de	https://www.facebook.com/hinews	ger	
693	idowa.de	https://www.facebook.com/idowa	ger	
694	inFranken.de	https://www.facebook.com/inFranken	ger	
695	inSüdthüringen	https://www.facebook.com/insuedthueringen	ger	
696	junge Welt	https://www.facebook.com/junge.welt	ger	
697	klagemauer.TV	https://www.facebook.com/klagemauerTV	ger	Questionable
698	kreiszeitung.de	https://www.facebook.com/kreiszeitung.de	ger	
699	legitim.ch	https://www.facebook.com/janwalter1983	ger	Questionable
700	mittelhessen.de	https://www.facebook.com/mittelhessen.de	ger	

	Page	Facebook	Language	Reliability
701	netzpolitik.org	https://www.facebook.com/netzpolitik	ger	
702	neues deutschland	https://www.facebook.com/nd.aktuell	ger	
703	news.de	https://www.facebook.com/News.de	ger	
704	news38.de	https://www.facebook.com/news38.de.funke	ger	
705	nordbayern.de	https://www.facebook.com/nordbayern.de	ger	
706	noz.de	https://www.facebook.com/neueoz	ger	
707	nrw-aktuell.tv	https://www.facebook.com/nrwaktuelltv	ger	
708	ntv Nachrichten	https://www.facebook.com/ntvNachrichten	ger	
709	op-online.de	https://www.facebook.com/oponline.de	ger	
710	presseportal	https://www.facebook.com/presseportal	ger	
711	rbb24	https://www.facebook.com/rbb24.de	ger	Reliable
712	scinexx.de - Das Wissensmagazin	https://www.facebook.com/scinexx	ger	
713	shz.de - Nachrichten aus Schleswig-Holstein	https://www.facebook.com/shzonline	ger	
714	stern	https://www.facebook.com/stern	ger	Reliable
715	svz.de - Nachrichten aus Mecklenburg-Vorpommern	https://www.facebook.com/svzonline	ger	
716	swissinfo	https://www.facebook.com/swissinfo	ger	
717	t-online	https://www.facebook.com/tonline	ger	Reliable
718	t3n Magazin	https://www.facebook.com/t3nMagazin	ger	
719	tagesschau	https://www.facebook.com/tagesschau	ger	
720	taz	https://www.facebook.com/taz.kommune	ger	
721	tz München	https://www.facebook.com/tzmuenchen	ger	
722	volksfreund.de	https://www.facebook.com/Volksfreund	ger	
723	watson.de	https://www.facebook.com/watsonDeutschland	ger	Reliable
724	Ärzte Zeitung	https://www.facebook.com/AerzteZeitung	ger	
725	100 Giorni da Leoni	https://www.facebook.com/100GiorniDaLeoni	ita	Questionable
726	ANSA.it	https://www.facebook.com/AgenziaANSA	ita	Reliable
727	AbruzzoLive	https://www.facebook.com/abruzzolive.it	ita	
728	Adnkronos	https://www.facebook.com/AgenziaAdnKronos	ita	
729	Agi	https://www.facebook.com/Notizie.Agi	ita	
730	AgriFoodToday	https://www.facebook.com/AgriFoodToday	ita	Reliable
731	Agrigentonotizie.it	https://www.facebook.com/profile.php?id=100063604569610	ita	
732	Aleteia Italiano	https://www.facebook.com/Aleteiait	ita	
733	Alguer.it	https://www.facebook.com/Alguer.it	ita	
734	Alto Adige	https://www.facebook.com/AltoAdigequotidiano	ita	
735	AmbienteBio	https://www.facebook.com/AmbienteBio	ita	Questionable
736	Amica	https://www.facebook.com/amicamagazine	ita	
737	AndriaLive.it	https://www.facebook.com/AndriaLive.it	ita	
738	Associazione Nazionale Papaboys	https://www.facebook.com/Papaboys	ita	
739	Associazione Rousseau	https://www.facebook.com/associazionerousseau	ita	Reliable
740	AvellinoToday	https://www.facebook.com/avellinotoday	ita	
741	Avvenire	https://www.facebook.com/avvenire.it	ita	
742	BUTAC - Bufale un tanto al chilo	https://www.facebook.com/BufaleUnTantoAlChilo	ita	
743	BZ News 24	https://www.facebook.com/bznews24	ita	
744	BariToday	https://www.facebook.com/BariToday.it	ita	
745	Beppe Grillo	https://www.facebook.com/beppegrillo.it	ita	
746	BisceglieLive.it	https://www.facebook.com/BisceglieLive	ita	
747	Blasting News	https://www.facebook.com/BlastingNews	ita	
748	Blitz Quotidiano	https://www.facebook.com/blitzquotidiano	ita	
749	BlogSicilia	https://www.facebook.com/blogsicilia	ita	Reliable
750	Blogo	https://www.facebook.com/BlogoNews	ita	
751	BolognaToday	https://www.facebook.com/BolognaToday	ita	
752	BresciaToday	https://www.facebook.com/BresciaToday	ita	Reliable
753	BrindisiReport.it	https://www.facebook.com/profile.php?id=100042394793461	ita	
754	CICAP	https://www.facebook.com/cicap.org	ita	
755	Caffeina	https://www.facebook.com/Caffeinafestival	ita	
756	Cagliari Live Magazine & Web Tv	https://www.facebook.com/cagliarilive	ita	Reliable
757	CasaDelSole.TV	https://www.facebook.com/casadelsole.tv	ita	Questionable
758	Caserta News	https://www.facebook.com/profile.php?id=100063551352139	ita	
759	CastelvetranoSelinunte.it	https://www.facebook.com/castelvetranoselinunte	ita	
760	CataniaToday	https://www.facebook.com/profile.php?id=100064405223319	ita	
761	CesenaToday	https://www.facebook.com/cesenatoday	ita	
762	ChietiToday	https://www.facebook.com/ChietiToday	ita	
763	Ci Siamo	https://www.facebook.com/CiSiamo.info	ita	Questionable
764	Claudio Messora	https://www.facebook.com/byoblu	ita	Questionable
765	Contro TV	https://www.facebook.com/controtvmedia	ita	Questionable
766	ControCopertina	https://www.facebook.com/Controcopertina	ita	Questionable
767	Contropiano.org	https://www.facebook.com/contropiano	ita	Questionable

	Page	Facebook	Language	Reliability
768	Corriere Adriatico.it	https://www.facebook.com/corriereadriatico.it	ita	
769	Corriere Romagna	https://www.facebook.com/corriereromagna	ita	
770	Corriere dell'Umbria	https://www.facebook.com/corrumbria	ita	
771	Corriere della Sera	https://www.facebook.com/corrieredellasera	ita	
772	Corriere delle Alpi	https://www.facebook.com/corrieredellealpi	ita	
773	Corriere di Arezzo	https://www.facebook.com/profile.php?id=100063923464154	ita	
774	Corriere di Rieti	https://www.facebook.com/corrieredirieti	ita	
775	Corriere di Siena	https://www.facebook.com/corrieredisiena	ita	
776	Corriere di Viterbo	https://www.facebook.com/corrierediviterbo	ita	
777	Corvelva	https://www.facebook.com/corvelva	ita	Questionable
778	CronacaQui	https://www.facebook.com/CronacaQui	ita	
779	Cronache Maceratesi	https://www.facebook.com/cronachemaceratesi	ita	
780	Cronache della Campania	https://www.facebook.com/cronachedellacampania	ita	
781	Curioctopus.it	https://www.facebook.com/Curioctopus.it	ita	
782	Curiosando	https://www.facebook.com/CuriosityworldIT	ita	
783	Curiosando si impara	https://www.facebook.com/curiosandosimpara	ita	
784	DINAMOpress	https://www.facebook.com/dinamopress	ita	
785	Dagospia	https://www.facebook.com/DagospiaUfficiale	ita	Questionable
786	DiariodelWeb.it	https://www.facebook.com/diariodelweb.it	ita	Reliable
787	Diâ€¢Lei	https://www.facebook.com/dilei.italiaonline	ita	
788	Domani	https://www.facebook.com/domanigiornale	ita	
789	Dottore, ma e vero che	https://www.facebook.com/dottoremaeverochefnomceo	ita	Reliable
790	Elbareport Quotidiano	https://www.facebook.com/elbareport.quotidiano	ita	
791	Epoch Times Italia	https://www.facebook.com/EpochTimesItalia	ita	Questionable
792	Eunews.it	https://www.facebook.com/Eunews.it	ita	Reliable
793	Europa Today	https://www.facebook.com/Europatoday.it	ita	Reliable
794	Facta	https://www.facebook.com/Facta.news	ita	Reliable
795	Famiglia Cristiana	https://www.facebook.com/famigliacristiana	ita	
796	Fanpage.it	https://www.facebook.com/fanpage.it	ita	Reliable
797	Fidelity News	https://www.facebook.com/fidelitynews	ita	
798	FirenzeToday	https://www.facebook.com/FirenzeToday	ita	
799	Focus	https://www.facebook.com/focus.it	ita	Reliable
800	Focus Junior	https://www.facebook.com/focusjuniorrivista	ita	
801	FoggiaToday	https://www.facebook.com/FoggiaToday	ita	Reliable
802	Fondazione AIRC per la Ricerca sul Cancro	https://www.facebook.com/AIRC.it	ita	
803	Forbes	https://www.facebook.com/forbes	ita	
804	ForliToday	https://www.facebook.com/forlitoday	ita	
805	Formiche	https://www.facebook.com/formichenews	ita	
806	Foto che ti daranno fastidio	https://www.facebook.com/fotocheturbano	ita	
807	FrosinoneToday	https://www.facebook.com/frosinonetoday	ita	
808	GQ Italia	https://www.facebook.com/gqitalia	ita	
809	Gay.it	https://www.facebook.com/gaypuntoit	ita	
810	Gazzetta di Mantova	https://www.facebook.com/GazzettadiMantova	ita	
811	Gazzetta di Parma	https://www.facebook.com/gazzettadiparma	ita	
812	Genova24	https://www.facebook.com/Genova24	ita	
813	GenovaToday	https://www.facebook.com/genovatoday	ita	
814	Geopop	https://www.facebook.com/geologiapop	ita	
815	Gianluigi Paragone	https://www.facebook.com/gianluigi.paragone	ita	Questionable
816	Giornale di Brescia	https://www.facebook.com/giornaledibrescia	ita	
817	Giornale di Sicilia	https://www.facebook.com/giornaledisicilia	ita	
818	Giornalettismo	https://www.facebook.com/Giornalettismo	ita	
819	Gold WebTv	https://www.facebook.com/Goldwebtv.it	ita	
820	GreenMe	https://www.facebook.com/greenme.it	ita	Reliable
821	Grottaglie InRete	https://www.facebook.com/girgrottaglieinrete	ita	
822	GuardaCheVideo	https://www.facebook.com/guardachevideo	ita	
823	HuffPost Italia	https://www.facebook.com/HuffPostItalia	ita	Reliable
824	Humanitas Salute	https://www.facebook.com/humanitasalute	ita	
825	I Love Italia	https://www.facebook.com/loveitaly90	ita	
826	INAF	https://www.facebook.com/media.inaf	ita	
827	ISSalute	https://www.facebook.com/ISSalute	ita	
828	IVG.it	https://www.facebook.com/IVG.it	ita	
829	Il Centro - Quotidiano d'Abruzzo	https://www.facebook.com/IlCentroQuotidiano	ita	
830	Il Corriere della CittÃ	https://www.facebook.com/IlCorrieredellaCitta	ita	
831	Il Fatto Quotidiano	https://www.facebook.com/ilFattoQuotidiano	ita	
832	Il Foglio	https://www.facebook.com/ilfoglio	ita	
833	Il Gallo	https://www.facebook.com/redazione.ilgallo	ita	
834	Il Gazzettino	https://www.facebook.com/gazzettino.it	ita	

	Page	Facebook	Language	Reliability
835	Il Giornale	https://www.facebook.com/ilGiornale	ita	
836	Il Giornale di Vicenza.it	https://www.facebook.com/GdV.it	ita	
837	Il Giorno	https://www.facebook.com/IlGiorno	ita	
838	Il Granchio	https://www.facebook.com/ilgranchio.it	ita	
839	Il Grande Inganno DUE	https://www.facebook.com/grandeinganno.it	ita	Questionable
840	Il Mattino	https://www.facebook.com/ilmattino.it	ita	
841	Il Meridiano News	https://www.facebook.com/IlMeridianoNews	ita	
842	Il Messaggero	https://www.facebook.com/Messaggero.it	ita	
843	Il Piccolo	https://www.facebook.com/piccoloditrieste	ita	
844	Il Piccolo - Alessandria	https://www.facebook.com/ilpiccolodialessandria	ita	Reliable
845	Il Primato Nazionale - Mensile	https://www.facebook.com/ilprimatonatsionale	ita	Questionable
846	Il Resto del Carlino	https://www.facebook.com/IlRestoDelCarlino	ita	
847	Il Riformista	https://www.facebook.com/IlRiformistaGiornale	ita	
848	Il Sannio Quotidiano	https://www.facebook.com/IlSannioQuotidiano	ita	
849	Il Secolo XIX	https://www.facebook.com/IlSecoloXIX	ita	
850	Il Sole 24 ORE	https://www.facebook.com/ilsole24ore	ita	
851	Il Sussidiario.net	https://www.facebook.com/ilSussidiarionet	ita	
852	Il Telegrafo Livorno	https://www.facebook.com/iltelegrafolivorno	ita	
853	Il Tempo Quotidiano	https://www.facebook.com/iltempoquotidiano	ita	
854	Il Vaso di Pandora	https://www.facebook.com/ilvasodipandora2.0	ita	Questionable
855	Il sapere e' potere	https://www.facebook.com/profile.php?id=100057456857554	ita	Questionable
856	IlPescara	https://www.facebook.com/IlPescara.it	ita	
857	IlPiacenza	https://www.facebook.com/IlPiacenza.it	ita	
858	IlSicilia.it	https://www.facebook.com/ilsicilia.it	ita	Reliable
859	Imola Oggi	https://www.facebook.com/imolaoggi2	ita	Questionable
860	Infodifesa	https://www.facebook.com/infodifesa	ita	
861	Informare x Resistere	https://www.facebook.com/informareXresistere	ita	Questionable
862	Informazione.it - Notizie	https://www.facebook.com/Informazione.it	ita	Reliable
863	Infosannio	https://www.facebook.com/informazionequotidiana	ita	Questionable
864	InsideOver	https://www.facebook.com/insideoverworld	ita	Reliable
865	InterNapoli.it	https://www.facebook.com/InterNapoli.it	ita	
866	Internazionale	https://www.facebook.com/Internazionale	ita	
867	InvestireOggi.it	https://www.facebook.com/Investireoggi	ita	
868	Irpinianews.it	https://www.facebook.com/irpinianews	ita	
869	IsNews.it - Molise	https://www.facebook.com/isnews.it	ita	Reliable
870	Istituto Superiore di SanitÃ	https://www.facebook.com/ISS.social	ita	
871	Italia Feed	https://www.facebook.com/ItaliaFeed	ita	Questionable
872	ItaliaOggi	https://www.facebook.com/italiaoggi.it	ita	
873	Jeda News	https://www.facebook.com/JedaNews	ita	Questionable
874	L'Adige - Quotidiano indipendente del Trentino Alto Adige	https://www.facebook.com/giornaleladige	ita	
875	L'Ancora	https://www.facebook.com/settimanale.lancora	ita	
876	L'AntiDiplomatico	https://www.facebook.com/lantidiplomatico	ita	Questionable
877	L'Arena	https://www.facebook.com/LArena.it	ita	
878	L'Eco del Chisone	https://www.facebook.com/ecodelchisone	ita	
879	L'Eco di Bergamo	https://www.facebook.com/lecodibergamo	ita	
880	L'Espresso	https://www.facebook.com/espressonline	ita	
881	L'Osservatore Romano	https://www.facebook.com/ossromano	ita	
882	L'Unione Sarda	https://www.facebook.com/UnioneSarda	ita	
883	La Gazzetta del Mezzogiorno	https://www.facebook.com/lagazzettadelmezzogiorno.it	ita	
884	La Luce di Maria	https://www.facebook.com/LaLucediMaria	ita	
885	La Nazione	https://www.facebook.com/LaNazione	ita	Reliable
886	La Notizia	https://www.facebook.com/LANOTIZIAgiornale.it	ita	
887	La Nuova Bussola Quotidiana	https://www.facebook.com/lanuovabq	ita	Questionable
888	La Nuova Sardegna	https://www.facebook.com/lanuovasardegna	ita	
889	La Nuova di Venezia e Mestre	https://www.facebook.com/lanuovadiveneziaemestre	ita	Reliable
890	La Provincia di Como	https://www.facebook.com/profile.php?id=100063583779711	ita	
891	La Provincia di Cremona	https://www.facebook.com/laprovinciadicremona	ita	
892	La Provincia di Lecco	https://www.facebook.com/laprovinciadilc	ita	
893	La Provincia di Sondrio	https://www.facebook.com/profile.php?id=100063959464085	ita	
894	La Provincia pavese	https://www.facebook.com/laprovinciapavese	ita	
895	La Refubblica	https://www.facebook.com/larefubblica.it	ita	
896	La Stampa	https://www.facebook.com/lastampa.it	ita	
897	La Valsusa	https://www.facebook.com/lavalsusa	ita	
898	La VeritÃ	https://www.facebook.com/quotidianolaverita	ita	
899	La Voce	https://www.facebook.com/gruppolavoce	ita	
900	La Voce Del Trentino	https://www.facebook.com/La.Voce.Del.Trentino	ita	Questionable
901	La Voce del Patriota	https://www.facebook.com/lavocedelpatriota	ita	Questionable

	Page	Facebook	Language	Reliability
902	La Voce di Mantova	https://www.facebook.com/vocedimantova	ita	
903	LaPresse	https://www.facebook.com/LaPresseNews	ita	
904	LatinaToday	https://www.facebook.com/Latinatodaynotizielatina	ita	
905	Le Scienze	https://www.facebook.com/LeScienze	ita	
906	LeccePrima.it	https://www.facebook.com/LeccePrima	ita	
907	LeccoToday	https://www.facebook.com/LeccoToday	ita	
908	Left	https://www.facebook.com/LeftAvvenimenti	ita	
909	Leggo	https://www.facebook.com/qleggo	ita	
910	Lercio	https://www.facebook.com/lercio.it	ita	
911	Libero	https://www.facebook.com/liberonews	ita	Questionable
912	LifeGate	https://www.facebook.com/lifegate	ita	Reliable
913	Liguria Notizie	https://www.facebook.com/liguria.notizie	ita	
914	Limes, rivista italiana di geopolitica	https://www.facebook.com/limesonline	ita	
915	Linkiesta.it	https://www.facebook.com/linkiesta	ita	
916	LiveSicilia	https://www.facebook.com/LiveSicilia.it	ita	
917	LivornoToday	https://www.facebook.com/LivornoToday	ita	Reliable
918	Luna Nuova	https://www.facebook.com/editricelunanuova	ita	
919	MARSICALIVE	https://www.facebook.com/marsicalive	ita	
920	MF-Milano Finanza	https://www.facebook.com/milanofinanza	ita	
921	MSN.fr	https://www.facebook.com/MSN.fr	ita	
922	MarieClaire.it	https://www.facebook.com/MarieClaireItalia	ita	Questionable
923	Massimo Mazzucco	https://www.facebook.com/massimo.mazzucco.7	ita	Questionable
924	MedBunker	https://www.facebook.com/MedBunker	ita	
925	Medical Facts di Roberto Burioni	https://www.facebook.com/robertoburioniMD	ita	
926	Medicitalia	https://www.facebook.com/medicitalia	ita	
927	MeridioNews	https://www.facebook.com/MeridioNews	ita	Reliable
928	Messaggero Veneto	https://www.facebook.com/messaggeroveneto	ita	
929	MessinaToday	https://www.facebook.com/messinatoday.it	ita	Reliable
930	MeteoWeb	https://www.facebook.com/meteoweb	ita	
931	Metro News Italia	https://www.facebook.com/MetroNewsItalia	ita	
932	Metropolis Quotidiano	https://www.facebook.com/metropolisquotidiano	ita	
933	Michele Santoro	https://www.facebook.com/michelesantoropresenta	ita	
934	Milano CittÃ Stato	https://www.facebook.com/milanocittastato	ita	
935	MilanoToday	https://www.facebook.com/MilanoToday	ita	
936	Ministero della Salute	https://www.facebook.com/MinisteroSalute	ita	
937	ModenaToday	https://www.facebook.com/Modenatoday	ita	
938	MolfettaLive.it	https://www.facebook.com/MolfettaLive	ita	
939	Money.it	https://www.facebook.com/moneypuntoit	ita	Reliable
940	MonzaToday	https://www.facebook.com/MonzaToday	ita	
941	NanoTV	https://www.facebook.com/nanotvofficial	ita	
942	NapoliToday	https://www.facebook.com/NapoliToday	ita	Reliable
943	National Geographic	https://www.facebook.com/NatGeoDeutschland	ita	
944	NewNotizie.it	https://www.facebook.com/NewNotizie	ita	
945	Next quotidiano	https://www.facebook.com/nextquotidiano.it	ita	
946	Nicola Porro	https://www.facebook.com/NicPorro	ita	Questionable
947	No Islam Italia	https://www.facebook.com/NoIslamItalia	ita	Questionable
948	Nogeoingegneria	https://www.facebook.com/profile.php?id=100064815873387	ita	Questionable
949	Norba Online	https://www.facebook.com/norbaonline	ita	Reliable
950	Notizie.it	https://www.facebook.com/notizie.it	ita	
951	NovaraToday	https://www.facebook.com/NovaraToday	ita	
952	Nuovo Quotidiano di Puglia online	https://www.facebook.com/quotidianodipuglia	ita	
953	OGGI	https://www.facebook.com/oggisettimanale	ita	
954	OK Salute e Benessere	https://www.facebook.com/OkSalute	ita	
955	OM - Optimagazine	https://www.facebook.com/OptiMagazine	ita	Questionable
956	Oasi Sana - percorso olistico di benessere biologico e naturale	https://www.facebook.com/profile.php?id=100080187814322	ita	Questionable
957	Oggi Treviso	https://www.facebook.com/oggitreviso	ita	
958	Oltre.tv	https://www.facebook.com/oltretv	ita	Questionable
959	Open	https://www.facebook.com/Opengiornaleonline	ita	Reliable
960	Orizzonte Scuola	https://www.facebook.com/OrizzonteScuola.it	ita	
961	Ottopagine	https://www.facebook.com/Ottopagine	ita	
962	PadovaOggi	https://www.facebook.com/PadovaOggi	ita	
963	Pagella Politica	https://www.facebook.com/PagellaPolitica	ita	
964	PalermoToday	https://www.facebook.com/PalermoToday	ita	
965	Pandora TV	https://www.facebook.com/PANDORATV.IT	ita	Questionable
966	Panorama.it	https://www.facebook.com/panorama.it	ita	
967	ParmaToday	https://www.facebook.com/ParmaToday	ita	
968	PisaToday	https://www.facebook.com/PisaToday	ita	Reliable

	Page	Facebook	Language	Reliability
969	Positano Notizie	https://www.facebook.com/positanonotizia	ita	
970	Pressenza en castellano	https://www.facebook.com/pressenza	ita	Reliable
971	Prima Monza	https://www.facebook.com/primamonza.it	ita	
972	Pro Vita & Famiglia Onlus	https://www.facebook.com/provitaonlus	ita	Questionable
973	Query	https://www.facebook.com/queryonline	ita	
974	Qui Como	https://www.facebook.com/qui.como	ita	
975	Qui News Elba	https://www.facebook.com/QuiElba.it	ita	
976	Qui News Pisa	https://www.facebook.com/QuiNewsPisa.it	ita	Reliable
977	Qui News Valdera	https://www.facebook.com/QuiValdera.it	ita	Reliable
978	QuiCosenza.it	https://www.facebook.com/quicosenza	ita	
979	QuiFinanza	https://www.facebook.com/QuiFinanza	ita	
980	Quotidiano SanitÃ	https://www.facebook.com/QSanit	ita	
981	Quotidiano del Sud - L'Altravoce dell'Italia	https://www.facebook.com/quotidianodelsud	ita	
982	Quotidiano di Sicilia	https://www.facebook.com/QdS.it	ita	
983	Quotidianomolise.it	https://www.facebook.com/quotmolis	ita	
984	RIZA Benessere	https://www.facebook.com/RIZABenessere	ita	Reliable
985	ROBA DA DONNE	https://www.facebook.com/robadadonne	ita	
986	RTL 102.5	https://www.facebook.com/RTL102.5	ita	
987	RTM	https://www.facebook.com/radiortm.it	ita	
988	Rainews.it	https://www.facebook.com/rainews.it	ita	
989	Rassegne Italia	https://www.facebook.com/rassegneitalia	ita	Questionable
990	RavennaToday	https://www.facebook.com/ravennatoday	ita	
991	Ravennawebtv	https://www.facebook.com/ravennawebtv	ita	
992	Redazione Agenpress	https://www.facebook.com/agenpress	ita	Questionable
993	ReggioToday	https://www.facebook.com/reggiotoday	ita	
994	RiminiToday	https://www.facebook.com/riminitoday	ita	
995	Rinnovabili.it	https://www.facebook.com/rinnovabiliit	ita	
996	Riviera Oggi	https://www.facebook.com/rivieraoggi.it	ita	
997	Riviera24	https://www.facebook.com/riviera24	ita	
998	Rivista Studio	https://www.facebook.com/rivistastudio	ita	
999	Rolling Stone Italia	https://www.facebook.com/rollingstoneitalia	ita	
1000	Roma - Il Giornale di Napoli	https://www.facebook.com/QuotidianoRoma	ita	
1001	RomaToday	https://www.facebook.com/romatoday	ita	
1002	Romagna Oggi	https://www.facebook.com/romagna.oggi	ita	
1003	Rumble	https://www.facebook.com/rumblevideo	ita	
1004	SAN FRANCESCO D'ASSISI	https://www.facebook.com/sanfrancescoassisi	ita	
1005	Sabato Sera	https://www.facebook.com/SabatoSeraSettimanale	ita	
1006	SalernoToday	https://www.facebook.com/SalernoToday	ita	Reliable
1007	Saluteinternazionale.info	https://www.facebook.com/saluteint	ita	
1008	SardiniaPost	https://www.facebook.com/SardiniaPost	ita	Reliable
1009	SaronnoNews	https://www.facebook.com/saronnonews	ita	Reliable
1010	Scenarieconomici.it	https://www.facebook.com/scenarieconomic	ita	Questionable
1011	Scienza in rete	https://www.facebook.com/scienzainrete	ita	Reliable
1012	Scienze Notizie	https://www.facebook.com/scienzenotizie	ita	
1013	Scuolainforma	https://www.facebook.com/scuolainforma	ita	
1014	Secolo d'Italia	https://www.facebook.com/secoloditalia	ita	
1015	Segnidalcielo - Segni di una Nuova Era	https://www.facebook.com/Segnidalcielo.it	ita	Questionable
1016	Silenzi e falsitÃ della stampa italiana	https://www.facebook.com/silenziefalsita	ita	
1017	Sky TG24	https://www.facebook.com/SkyTG24	ita	
1018	SondrioToday	https://www.facebook.com/ValtellinaToday	ita	
1019	Sputnik Italia	https://www.facebook.com/it.sputnik	ita	Questionable
1020	Stefano Montanari	https://www.facebook.com/stefanomontanari.net	ita	Questionable
1021	StopCensura	https://www.facebook.com/stopcensura.online	ita	Questionable
1022	TELEBARI	https://www.facebook.com/telebari	ita	
1023	TPI	https://www.facebook.com/thepostinternazionale	ita	
1024	Taranto BuonaSera	https://www.facebook.com/buonaserataranto	ita	
1025	Tele Club Italia	https://www.facebook.com/teleclubitalia.it	ita	
1026	Teleambiente	https://www.facebook.com/teleambiente	ita	
1027	Teleromagna	https://www.facebook.com/Teleromagna	ita	
1028	Tempi	https://www.facebook.com/tempi.it	ita	
1029	Tempostretto.it - Quotidiano online di Messina e Reggio Calabria	https://www.facebook.com/Tempostretto.it	ita	
1030	TermoliOnLine.it	https://www.facebook.com/TermoliOnLine	ita	
1031	Termometro Politico	https://www.facebook.com/termometropolitico	ita	
1032	Tg La7	https://www.facebook.com/tgla7	ita	
1033	Tgcom24	https://www.facebook.com/tgcom24	ita	
1034	The Saker - Italia	https://www.facebook.com/SakerItalia	ita	Questionable
1035	The Social Post	https://www.facebook.com/thesocialpost.it	ita	

	Page	Facebook	Language	Reliability
1036	The Vision	https://www.facebook.com/thevisioncom	ita	
1037	Tiscali.it	https://www.facebook.com/Tiscali	ita	
1038	Today.it	https://www.facebook.com/Today.Notizie	ita	
1039	TorinoToday	https://www.facebook.com/TorinoToday	ita	
1040	Toscana Media News	https://www.facebook.com/ToscanaMediaNews	ita	Reliable
1041	Trentino	https://www.facebook.com/Trentin0	ita	
1042	TrentoToday	https://www.facebook.com/TrentoToday	ita	Reliable
1043	Tv2000	https://www.facebook.com/Tv2000it	ita	Reliable
1044	Ultim’ora.news	https://www.facebook.com/Ultimora.news.ufficiale	ita	Reliable
1045	Ultime Notizie Flash	https://www.facebook.com/UNFultimeNotizieFlash	ita	Reliable
1046	Umbria24	https://www.facebook.com/Umbria24	ita	
1047	Universo Mamma	https://www.facebook.com/UniversoMamma	ita	
1048	VICE Italia	https://www.facebook.com/vice.italia	ita	
1049	VITA non profit	https://www.facebook.com/VitaSocialContent	ita	
1050	Valigia Blu	https://www.facebook.com/valigiablu	ita	
1051	Vanity Fair Italia	https://www.facebook.com/vanityfairitalia	ita	
1052	VareseNews	https://www.facebook.com/varesenews	ita	
1053	Vatican News	https://www.facebook.com/vaticannews	ita	
1054	VeneziaToday	https://www.facebook.com/VeneziaToday	ita	
1055	Verona Sera	https://www.facebook.com/veronasera	ita	
1056	Vesuvio live	https://www.facebook.com/vesuviolive	ita	
1057	VicenzaToday	https://www.facebook.com/profile.php?id=100063507192683	ita	
1058	VideoAndria.com	https://www.facebook.com/VideoAndriaWebtv	ita	
1059	Vimeo	https://www.facebook.com/Vimeo	ita	
1060	Virgilio.it	https://www.facebook.com/Virgilio.Portale	ita	
1061	Visione TV	https://www.facebook.com/visionetv	ita	Questionable
1062	ViterboNews 24	https://www.facebook.com/viterbonews24	ita	
1063	Voce di Napoli	https://www.facebook.com/vocenapoli	ita	
1064	Wall Street Italia	https://www.facebook.com/wallstreetita	ita	
1065	Wired Italia	https://www.facebook.com/wireditalia	ita	
1066	affaritaliani.it	https://www.facebook.com/affaritaliani	ita	
1067	askanews	https://www.facebook.com/askanews	ita	
1068	globalist.it	https://www.facebook.com/globalist.it	ita	Questionable
1069	gonews.it	https://www.facebook.com/gonews.it	ita	
1070	greenreport.it	https://www.facebook.com/greenreport.it	ita	
1071	il Friuli	https://www.facebook.com/ilfriuli	ita	Questionable
1072	il Post	https://www.facebook.com/ilpost	ita	
1073	il manifesto	https://www.facebook.com/ilmanifesto	ita	
1074	il mattino di Padova	https://www.facebook.com/mattinodipadova	ita	
1075	ilcorrierino.com	https://www.facebook.com/ilcorrierino	ita	
1076	ilmamilio.it	https://www.facebook.com/il.mamilio	ita	
1077	la Repubblica	https://www.facebook.com/Repubblica	ita	
1078	la Sentinella del Canavese	https://www.facebook.com/lasentinelladelcanavese	ita	
1079	la tribuna di Treviso	https://www.facebook.com/latribunaditreviso	ita	Reliable
1080	lasicilia.it	https://www.facebook.com/lasiciliait	ita	
1081	meteo.it	https://www.facebook.com/meteoit	ita	
1082	salernonotizie.it	https://www.facebook.com/salernonotizie.it	ita	
